# Supplementary figures and images for: In-hospital mortality outcomes of favipiravir in patients with moderate to severe COVID-19 infection: An emulated target trial using real-world data from the largest field hospital in Thailand
Source: PLoS One. 2025 Jun 4;20(6):e0324903. doi: 10.1371/journal.pone.0324903 (PMC12136412; doi:10.1371/journal.pone.0324903)

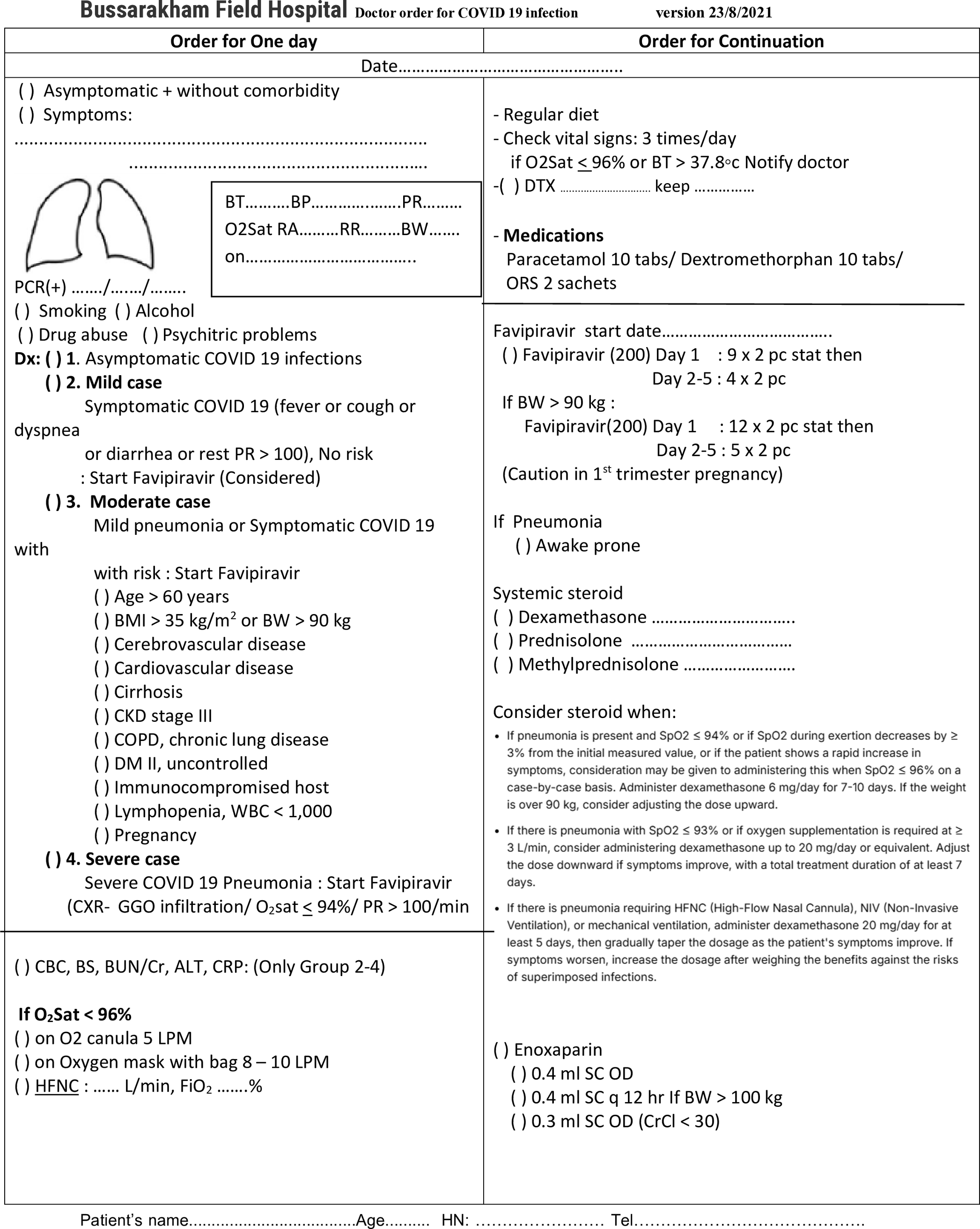

Supplement: S1 Fig — (TIF) [file pone.0324903.s001.tif]

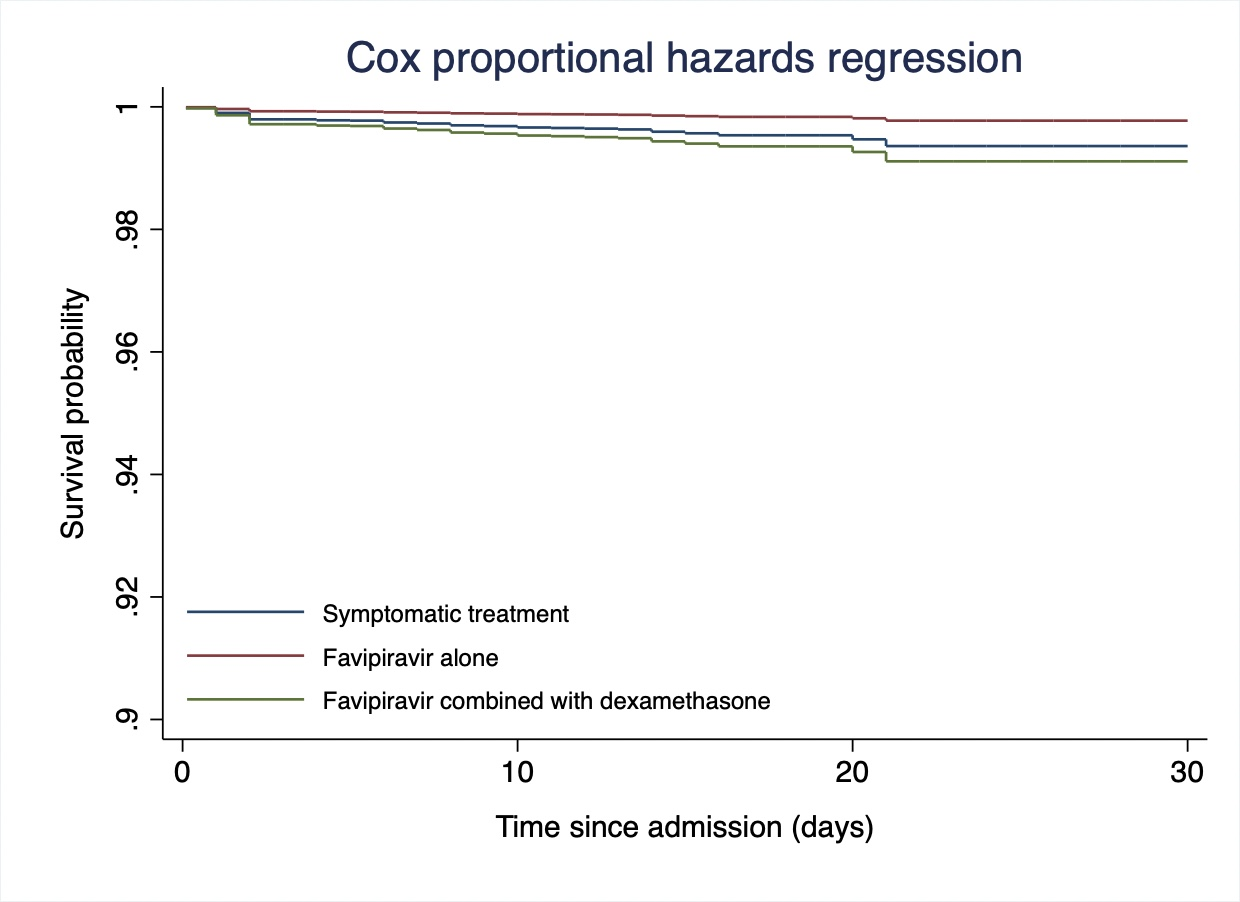

Supplement: S2 Fig — (TIF) [file pone.0324903.s002.tif]
