# Supplementary material for: In-hospital mortality outcomes of favipiravir in patients with moderate to severe COVID-19 infection: An emulated target trial using real-world data from the largest field hospital in Thailand
Source: PLoS One. 2025 Jun 4;20(6):e0324903. doi: 10.1371/journal.pone.0324903 (PMC12136412; doi:10.1371/journal.pone.0324903)
Supplement: S1 Table — (DOCX) [file pone.0324903.s003.docx]

**S1 Table.** Standardized difference (before vs after weighting)

| Standardized difference | Supportive treatment vs  Favipiravir alone | | Supportive treatment vs  Favipiravir combined with dexamethasone | | Favipiravir alone vs  Favipiravir combined with dexamethasone | |
| --- | --- | --- | --- | --- | --- | --- |
|  | Before | After | Before | After | Before | After |
| **Demographic** |  |  |  |  |  |  |
| Age above 60 years | **0.140** | 0.047 | **0.299** | **0.154** | **0.157** | **0.107** |
| Male | **-0.103** | **-0.156** | -0.037 | **-0.103** | 0.066 | 0.053 |
| BMI | **0.113** | 0.031 | **0.183** | 0.069 | 0.079 | 0.043 |
| Duration from PCR to admission | -0.073 | -0.081 | -0.098 | -0.083 | -0.026 | -0.002 |
| **Underlying disease** |  |  |  |  |  |  |
| Cerebrovascular disease | -0.036 | -0.001 | -0.018 | 0.012 | 0.018 | 0.011 |
| Cardiovascular disease | **0.117** | -0.007 | **0.134** | 0.063 | 0.018 | 0.056 |
| Cirrhosis |  | -0.015 | 0.084 | 0.041 | 0.084 | 0.054 |
| Chronic kidney disease | -0.008 | -0.036 | -0.008 | -0.001 | -0.001 | 0.009 |
| COPD | -0.021 | 0.014 | -0.040 | -0.028 | -0.018 | 0.008 |
| Diabetes mellitus | -0.084 | 0.1072 | **0.176** | **0.148** | **0.260** | 0.077 |
| Hypertension | 0.007 | 0.041 | **0.203** | **0.129** | **0.197** | 0.088 |
| Immunodeficiency | 0.085 | 0.026 | **0.103** | 0.035 | 0.025 | 0.019 |
| Obesity | 0.011 | 0.032 | 0.090 | 0.052 | 0.080 | 0.021 |
| **Vital sign** |  |  |  |  |  |  |
| Body temperature | -0.087 | 0.101 | 0.043 | 0.099 | **0.120** | 0.003 |
| Heart rate | **-0.168** | -0.005 | -0.022 | 0.002 | **0.147** | 0.008 |
| Systolic blood pressure | 0.094 | 0.030 | **0.185** | 0.085 | 0.088 | 0.053 |
| Diastolic blood pressure | **0.150** | -0.017 | **0.143** | 0.017 | -0.004 | 0.034 |
| Oxygen saturation | **0.462** | **0.227** | **0.333** | **0.140** | -0.136 | -0.088 |
| **Vaccine status** |  |  |  |  |  |  |
| Not receive any vaccine | -0.076 | 0.095 | -0.033 | 0.071 | 0.042 | -0.024 |
| Complete 1^st^ dose of vaccine | **0.112** | -0.063 | 0.093 | -0.039 | -0.019 | 0.023 |
| Complete 2^nd^ dose of vaccine | -0.053 | -0.079 | **-0.108** | -0.074 | -0.056 | 0.005 |
| **First respiratory support** |  |  |  |  |  |  |
| Room air | -0.048 | **-0.201** | **-0.889** | **-0.588** | **-0.835** | **-0.377** |
| Oxygen cannula | **0.150** | **0.256** | **0.874** | **0.504** | **0.707** | **0.243** |
| Oxygen mask | 0.017 | 0.027 | **0.134** | 0.045 | **0.119** | 0.072 |
| Oxygen high flow | **-0.189** | -0.091 | 0.051 | **0.184** | **0.238** | **0.269** |
