# Supplementary material for: In-hospital mortality outcomes of favipiravir in patients with moderate to severe COVID-19 infection: An emulated target trial using real-world data from the largest field hospital in Thailand
Source: PLoS One. 2025 Jun 4;20(6):e0324903. doi: 10.1371/journal.pone.0324903 (PMC12136412; doi:10.1371/journal.pone.0324903)
Supplement: S2 Table — (DOCX) [file pone.0324903.s004.docx]

**S2 Table.** Sensitivity analysis among patients with more severe characteristics

| **Treatment strategies among subgroup of patients** | **RMST** | | **Adjusted RMST Difference compared to ST** | | | **Adjusted RMST Difference compared to FPV alone** | | |
| --- | --- | --- | --- | --- | --- | --- | --- | --- |
|  | **Mean (days)** | **95% CI** | **Mean (days)** | **95% CI** | **p-value** | **Mean (days)** | **95% CI** | **p-value** |
| **Moderate-to-severe** |  |  |  |  |  |  |  |  |
| ST | 26.88 | 24.53,29.22 | - | - | - | - | - | - |
| FPV alone | 29.42 | 29.13,29.70 | 2.54 | 0.10,4.98 | **0.041** | - | - | - |
| FPV with Dexa | 29.71 | 29.51,29.91 | 2.83 | 0.81,4.86 | **0.006** | 0.29 | -0.91,1.50 | 0.632 |
| **Patients with hypoxia** |  |  |  |  |  |  |  |  |
| ST | 24.01 | 19.69,28.33 | - | - | - | - | - | - |
| FPV alone | 29.07 | 28.51,29.63 | 5.06 | 0.81,9.32 | **0.020** | - | - | - |
| FPV with Dexa | 29.53 | 29.19,29.88 | 5.53 | 0.37,10.68 | **0.036** | 0.46 | -0.25,1.17 | 0.201 |
| **Male patients** |  |  |  |  |  |  |  |  |
| ST | 26.02 | 23.61,28.43 | - | - | - | - | - | - |
| FPV alone | 29.06 | 28.70,29.43 | 3.04 | 1.02,5.07 | **0.003** | - | - | - |
| FPV with Dexa | 29.55 | 29.28,29.81 | 3.53 | 1.16,5.89 | **0.003** | 0.48 | -0.70,1.67 | 0.424 |
| **Patients with age > 60** |  |  |  |  |  |  |  |  |
| ST | 26.48 | 23.09,29.87 | - | - | - | - | - | - |
| FPV alone | 28.88 | 28.34,29.43 | 2.40 | -1.32,6.11 | 0.206 | - | - | - |
| FPV with Dexa | 29.09 | 28.55,29.65 | 2.62 | -2.14,7.37 | 0.281 | 0.22 | -0.41,0.84 | 0.498 |
| **Patients without any vaccine** |  |  |  |  |  |  |  |  |
| ST | 28.26 | 27.02,29.31 | - | - | - | - | - | - |
| FPV alone | 29.57 | 29.35,29.78 | 1.31 | 0.26,2.36 | **0.014** | - | - | - |
| FPV with Dexa | 29.74 | 29.58,29.89 | 1.48 | -1.45,4.41 | 0.323 | 0.17 | -0.22,0.56 | 0.390 |

**Abbreviations:** CI, confidence interval; Dexa, dexamethasone; FPV, favipiravir; RMST, restricted mean survival time; ST, standard care.
