# Supplementary material for: In-hospital mortality outcomes of favipiravir in patients with moderate to severe COVID-19 infection: An emulated target trial using real-world data from the largest field hospital in Thailand
Source: PLoS One. 2025 Jun 4;20(6):e0324903. doi: 10.1371/journal.pone.0324903 (PMC12136412; doi:10.1371/journal.pone.0324903)
Supplement: S3 Table — (DOCX) [file pone.0324903.s005.docx]

**S3 Table.** Studies investigated mortality outcome of favipiravir in hospitalized moderate to critical COVID-19 patients.

| **Study, (Country)** | **Study design** | **Population** | **Intervention** | | **Outcome measurement** | **Mortality outcomes (n)** |
| --- | --- | --- | --- | --- | --- | --- |
|  |  |  | **FPV arms** | **Compared arms** |  |  |
| **Clinical trials** | |  |  |  |  |  |
| Bosaeed, 2021  (Saudi arabia) | Multicenter, open-label, RCT | Hospitalized moderate to severe patients with RT-PCR confirmed COVID-19 | **FPV + SOC + HCQ**  (FPV 1800 mg BID on first day followed by 800 mg BID for up to 10 days and HCQ 400 mg BID on day 1 and then 200 mg BID for 5 days) | **SOC+antivirals**  (based on local protocol) | - Time to clinical improvement  - Progress in clinical status  - Requirement of ICU admission or mechanical ventilation within 14 days.  - **28- and 90-day all-cause mortality** | 14 (12.61%) died in FPV; 15 (13.76%) in control (p = 0.91) |
| Chen, 2021  (China) | Prospective, multicenter, open-label, RCT | Hospitalized patients with COVID-19 pneumonia (moderate to severe) | **FPV + SOC** (1600 mg BID on first day followed by 600 mg BID for 7 days) | **Arbidol + SOC**  (Arbidol 200 mg TID for 7 days) | - Clinical recovery rate at 7 days  - Latency to pyrexia relief  - Latency to cough relief  - The rate  of AOT or NMV/MV  - **All-cause mortality**  - Dyspnea  - Rate of  respiratory failure | No deaths during the trial. |
| Luo, 2021  (China) | Single center, open-label, RCT | Hospitalized patients  with RT-PCR confirmed COVID-19 (moderate to critical) | **FPV + SOC**  (FPV at 1600 mg or 2200 mg, followed by 600 mg three times a day, not over 14 days) | **(1) Baloxavir marboxil** **+ SOC**  **(2) SOC**  (Either LPV/r 400  mg/100 mg BID or darunavir/ cobicistat 800 mg/150 mg QD and arbidol 200 mg TID) | - % subjects with viral negative by Day 14  - Time to improvement  - % subjects with viral negative by Day 7  - Mechanical ventilation  - ICU admission  - **All-cause mortality** | No deaths during the trial. |
| Shenoy, 2021  (Kuwait) | Multicenter, phase 3, double-blind, RCT | Hospitalized moderate-severe patients with RT-PCR confirmed COVID-19 | **FPV + SOC**  (FPV 1800 mg BID on first day followed by 800 mg BID for up to 10 days) | **placebo+SOC** | - Time to resolution of hypoxia  - Time to discharge  - **Mortality** | 14 (8.0%) in FPV; 11 (6.2%) in placebo  (p=0.54) |
| Solaymani-Dodaran, 2021  (Iran) | Multicenter, open-label, RCT | Hospitalized COVID-19 patients with RT-PCR confirm & pneumonia (moderate to severe) | **FPV + SOC + HCQ**  (FPV 1600  mg stat and then 600 mg every 8 h + HCQ 200 mg BID for 1 week) | **LPV/r + SOC +HCQ**  (single dose of  HCQ 400 mg followed by 100 + 400 LPV/r BID for 1 week) | - Number of ICU admissions  - Duration in hospital  **- In-hospital mortality**  - Time to clinical recovery  - Changes in SpO2 | 26 (13.7%) in FPV, and 21 (11.5%) in LPV/r (P =0.49) |
| Tabarsi, 2021  (Iran) | RCT | Hospitalized COVID-19 patients with RT-PCR confirm (moderate to severe) | **FPV**  (1600 mg BID on first day followed by 600 mg BID for up to 6 days) | **LPV/r**  (200/50 mg BID for 1 week) | - Changes in  baseline clinical symptoms  - ICU admission  - Length of ICU stay  - Need for anti-inflammatory agents  - Changes in baseline  radiological status  - Adverse drug reactions  - Length of hospitalization  **- Mortality** | 3 (9.37%) in FPV; 4 (13.33%) in LPV/r  (P = 0.463) |
| Kamali, 2023  (Iran) | RCT | Hospitalized COVID-19 patients with RT-PCR confirm (moderate to severe mean SpO2 <80%) | **FPV + SOC**  (600 mg BID for up to 7 days) | **SOC** | **- Mortality rate**  - Levels of SpO2  - Length of hospitalization  - Length of ICU stay | 7 (14%) in FPV; 30 (63.8) in control (p=0.0001) |
| **Observational study** | |  |  |  |  |  |
| Al-Muhsen, 2022  (Saudi Arabia) | Prospective, multicenter observational study | Hospitalized patients  with RT-PCR confirmed COVID-19 (moderate to severe) | **FPV**  (Either 1,800 or 1,600 mg BID on first day,  followed by 800 or 600 mg BID) | **Non-FPV and SOC** | **- All-cause mortality**  - Length of hospital stay | Adjusted HR 3.47 (95% CI, 1.24- 9.71; P=0.018) |
| Mutair, 2022  (Saudi Arabia) | Multicenter, retrospective cohort. Patients received FPV were extracted and matched controls were selected based on age, BMI | Critically ill COVID-19 patients admitted to ICU | **FPV with supplemental therapies** including Corticosteroids, Tocilizumab, Remdesivir, Chloroquine, Azithromycin  (Dosage was not reported) | **Other antimicrobial medications with supplemental therapies** including Corticosteroids, Tocilizumab, Remdesivir, Chloroquine, Azithromycin | **- Overall survival outcome**  - Hospital stay | 119 (44.2%) in FPV; 128 (47.6%) in control (P=0.4) |
| Sulaiman, 2023  (Saudi Arabia) | Retrospective cohort with PS match (1:1) | Critically ill patients with COVID-19 admitted to ICU | **FPV**  (median loading dose of 3600 mg/day  , and a median maintenance dose of 1600 mg/day, the median duration of therapy was 8 days) | **Non-FPV** | **- In-hospital mortality**  - Mechanical ventilation duration  - 30-day mortality  - Length of ICU stay  - Length of hospital stay  - Complications during the stay  - Laboratory investigation | **In-hospital mortality:** 47 (65.3%) in FPV; 32 (43.8%) in non-FPV (P=0.009)  HR 1.17 (95%CI, 0.73-1.87, P=0.51) |
| Chavalertsakul, 2024  (Thailand) | Retrospective cohort with PS match | COVID-19 patients with pneumonia admitted to ICU  (moderate to severe) | **FPV**  (1600 mg BID on first day followed by 600 mg BID for up to 10 days) | **Remdesivir**  (200 mg IV on first day, followed by a daily IV dose of 100 mg for up to 9 days) | **- 29-day Mortality outcomes**  - Length of hospital / ICU stay  - Recovery rate at 15 days | 28 (27.5%) in FPV; 26 (25.5%) in remdesivir  Adjusted HR 0.72 (95% CI, 0.41 to 1.25) |
| Srisubat, 2023 (Thailand) | Prospective Real-world data | Symptomatic patients with mild, moderate, and severe | **FPV** | No anti-viral agents | **- 28-day mortality risk** | 16 (1.29) in moderate; 129 (27%) in severe cases  RR = 0.72 (95% CI 0.58–0.91 P = 0.006) |
| Our study | Retrospective cohort with emulation of target trial | Hospitalized patients  with RT-PCR confirmed COVID-19 (moderate to severe) | **(1) FPV + Dexa + SOC**  (FPV 1800 mg BID on first day followed by 800 mg BID for up to 14 days;  Dexa 20 mg loading dose, then 5 mg daily for 5-7 days)  **(2) FPV + SOC** | **SOC** | - In-hospital mortality within 30 days | **Restricted mean survival time** in (1) **FPV + Dexa + SOC** 29.68 days (95% CI: 29.52, 29.84);  **(2) FPV + SOC** 29.46 days (95% CI: 29.22, 29.71) and **SOC** 28.14 days (95% CI: 26.51, 29.76) |

**Abbreviations:** BID, twice daily; CI, confidence interval; CT, computed tomography; dexa, dexamethasone; FPV, Favipiravir; HCQ, hydroxychloroquine; HR, hazard ratio; ICU, intensive care unit; IU, international unit; IV, intraveneous; LPV/r, Lopinavir/ Ritonavir; PS, propensity score; QD, once a day; RCT, randomized controlled trial; RT-PCR, reverse transcription polymerase chain reaction; SOC, standard of care; SpO2, Oxygen saturation; TID, three times a day
