# Supplementary material for: In-hospital mortality outcomes of favipiravir in patients with moderate to severe COVID-19 infection: An emulated target trial using real-world data from the largest field hospital in Thailand
Source: PLoS One. 2025 Jun 4;20(6):e0324903. doi: 10.1371/journal.pone.0324903 (PMC12136412; doi:10.1371/journal.pone.0324903)
Supplement: Attachment — Submitted filename: Response_to_Reviewers.docx [file pone.0324903.s007.docx]

**Dear Editor and reviewers**

We very much appreciate the excellent and thorough review of our manuscript entitled “**In-hospital mortality outcome of favipiravir in patients with moderate to severe COVID-19 infection: an emulated target trial using real-world data from the largest field hospital in Thailand**”. We have addressed all comments, point-by-point, as attached.

**Sincerely,**

**Phichayut Phinyo, MD, PhD**

Department of Biomedical Informatics and Clinical Epidemiology,

Faculty of Medicine, Chiang Mai University,

110 Intawaroros Road, Si Phum, Muang, Chiang Mai, Thailand 50200,

**Reviewers' comments:**

**Reviewer's Responses to Questions**

**Comments to the Author**

1. Is the manuscript technically sound, and do the data support the conclusions?

The manuscript must describe a technically sound piece of scientific research with data that supports the conclusions. Experiments must have been conducted rigorously, with appropriate controls, replication, and sample sizes. The conclusions must be drawn appropriately based on the data presented.

Reviewer #1: Yes

Reviewer #2: No

2. Has the statistical analysis been performed appropriately and rigorously?

Reviewer #1: Yes

Reviewer #2: Yes

3. Have the authors made all data underlying the findings in their manuscript fully available?

The [PLOS Data policy](http://track.editorialmanager.com/CL0/http:%2F%2Fwww.plosone.org%2Fstatic%2Fpolicies.action%23sharing/1/010f01938f6793ff-a91a4e0c-66e8-4865-889c-dbdc09fbf870-000000/lFSlrXTK_B6a95lBWWyvCvTqcvA8P5aA1vXPmZ0jL3E=187) requires authors to make all data underlying the findings described in their manuscript fully available without restriction, with rare exception (please refer to the Data Availability Statement in the manuscript PDF file). The data should be provided as part of the manuscript or its supporting information, or deposited to a public repository. For example, in addition to summary statistics, the data points behind means, medians and variance measures should be available. If there are restrictions on publicly sharing data—e.g. participant privacy or use of data from a third party—those must be specified.

Reviewer #1: Yes

Reviewer #2: No

4. Is the manuscript presented in an intelligible fashion and written in standard English?

PLOS ONE does not copyedit accepted manuscripts, so the language in submitted articles must be clear, correct, and unambiguous. Any typographical or grammatical errors should be corrected at revision, so please note any specific errors here.

Reviewer #1: Yes

Reviewer #2: Yes

5. Review Comments to the Author

Please use the space provided to explain your answers to the questions above. You may also include additional comments for the author, including concerns about dual publication, research ethics, or publication ethics. (Please upload your review as an attachment if it exceeds 20,000 characters)

**Reviewer #1: Dear Authors**

Thank you for your effort in this study.
The manuscript titled "In-hospital mortality outcome of favipiravir in patients with moderate to severe COVID-19 infection: an emulated target trial using real-world data from the largest field hospital in Thailand" evaluates the efficacy of favipiravir, with or without dexamethasone, in reducing in-hospital mortality among moderate to severe COVID-19 patients. Using a target trial emulation framework on data from over 3,000 patients in Thailand's largest field hospital, the study finds no significant improvement in 30-day survival outcomes for patients treated with favipiravir (alone or combined with dexamethasone) compared to those receiving standard symptomatic care.

The study highlights limitations such as residual confounding and the retrospective design, yet it contributes to the growing evidence questioning favipiravir’s role in treating severe COVID-19. It underscores the need for more rigorous prospective trials and suggests that favipiravir may not be essential in clinical practice for such cases.

While the study is well-conceived and executed, several areas require clarification, expanded discussion, and improved presentation to maximize its impact and clarity.

**My comments:

1- Abstract**

**Strengths:**
- The abstract is concise and provides a clear overview of the study’s aims, methods, results, and conclusions.
- It effectively communicates the novelty of the approach (emulated target trial) and its primary finding (no significant difference in mortality outcomes across treatment groups).

**Criticisms:**
- The p-values mentioned for the survival outcomes (e.g., p-value 0.479, 0.366) do not convey a strong interpretative value to general readers and could be omitted for brevity.
- The conclusion could better emphasize the implications of these findings for clinical guidelines, particularly in contexts where favipiravir is still widely used.

**Suggestions for Improvement:**
- Replace some numerical details with a more narrative style to improve accessibility (e.g., “no significant mortality difference was observed” instead of detailed p-values).
- Add a statement on the study’s real-world relevance to emphasize its significance.

**--------------------------------------------------------------**

**Response**

Thank you for your suggestion.

I have revised the abstracted and information related to the reviewers’ comment as follows:

**Abstract**

**Background:** Favipiravir, an antiviral agent, has been widely used to treat COVID-19 due to potential mechanism of action despite limited evidence of its efficacy in moderate to severe cases.

**Aim:** This study aimed to evaluate the efficacy of favipiravir in improving in-hospital mortality outcomes in moderate to severe COVID-19 patients through an emulation of a target trial.

**Methods:** We emulated a target trial using observational data from Bussarakham field hospital, Thailand during May 14 to September 20, 2021. Patients were categorized into three groups: those receiving favipiravir with dexamethasone (FPV with dexa), favipiravir alone (FPV), and symptomatic treatment (ST). In-hospital mortality at 30 days was the primary outcome.

**Results:** From 18,184 patients admitted to the hospital, a total of 3,193 moderate to severe COVID-19 cases were included. Of these, 2,256 (70.65%) received FPV with dexa, 828 (25.93%) received FPV, and 109 (3.41%) received ST. The restricted mean survival times for FPV with dexa was 29.68 days (95% CI: 29.52, 29.84), FPV was 29.46 days (95% CI: 29.22, 29.71), and ST was 28.14 days (95% CI: 26.51, 29.76). Only FPV showed marginal significant difference when compared to ST. However, there was a trend in prolonging survival time in FPV with dexa and the results were more pronounced in patients with severe and hypoxic groups.

**Conclusion:** Our emulated target trial suggests favipiravir, especially with dexamethasone, offers modest survival benefit in moderate to severe COVID-19, particularly in hypoxic patients. It supports favipiravir as a practical antiviral in setting where other antivirals are not available. Further controlled studies are needed to confirm its role alongside standard corticosteroid therapy.

**Keywords:** Favipiravir, COVID-19, In-hospital mortality, Target trial emulation,

**2- Introduction

Strengths:**
- Provides a detailed background on the role of favipiravir and its widespread use during the pandemic.
- Highlights the gap in evidence for its efficacy in moderate to severe COVID-19 cases, setting a strong rationale for the study.

**Criticisms:**
- Redundancy in describing the historical context of favipiravir’s use and the delta wave pandemic. While informative, some details could be streamlined.
- Limited critical analysis of conflicting evidence regarding favipiravir’s efficacy from past studies.

**Suggestions for Improvement:**
- Condense the description of favipiravir’s history to focus on its relevance to Thailand’s healthcare system.
- Introduce a clearer comparison of existing studies, highlighting where this study fills a knowledge gap.

**--------------------------------------------------------------**

**Response**

Thank you very much for the suggestion. We have revised our introduction as follows:

“Coronavirus disease 2019 (COVID-19) is a highly transmissible, severe acute respiratory illness caused by the coronavirus SARS-CoV- presenting a significant challenge to global healthcare systems and prompting extensive investigations into several antiviral agents [1, 2]. In Thailand, as in other countries, favipiravir was rapidly adopted as a first-line antiviral treatment for COVID-19 following its emergency authorization in 2020. [3-6] . This drug was included in the Thai Ministry of Public Health (MoPH) guidelines and widely used during the peak of the pandemic, particularly in response to the third phase that was driven by the delta variant in 2021 [7]. This variant significantly increased the number of patients requiring hospitalization, associated with higher mortality, and transmissibility [8, 9]. The surge in cases led to the establishment of Thailand’s largest national field hospital to manage patient overflow and ease the burden on healthcare facilities.

Despite its widespread use, favipiravir’s clinical efficacy in COVID-19 remains controversial [1, 10]. Several clinical trials focused primarily on mild COVID-19 cases, where favipiravir's effect is minimal, as most patients naturally recover with supportive care [11, 12]. However, in moderate to severe patients who are facing higher risks of respiratory failure and complications, antiviral and anti-inflammatory therapies may play a crucial role in improving outcomes [13, 14]. In addition, the study from RECOVERY trial demonstrated that dexamethasone, a potent anti-inflammatory steroid, reduced 28-day mortality in COVID-19 patients but not among those receiving no respiratory support [15]. Therefore, during the pandemic wave, the Thai MoPH guideline suggested the use of favipiravir in combination dexamethasone in patients with moderate to severe symptom.

In Thailand, studies investigated the use of favipiravir in COVID-19 patients are partly limited. Studies by Sirijatuphat et al. [16] and Siripongboonsitti et al. [17] compare efficacy of favipiravir to control treatment and found that favipiravir showed improvements on clinical outcomes in mild to moderate COVID-10 patients. Rattanaumpawan et al. [18]reported the favipiravir’s effectiveness in reducing hospital stays among hospitalized patients, though its impact on mortality remained unclear. Furthermore, a real-world study during the Delta variant wave indicated that favipiravir monotherapy reduced the 28-day mortality risk in severe COVID-19 (Relative risk = 0.72 (95% CI 0.58–0.91; P = 0.006) but not in mild and moderate patients [19]. The results are still inconclusive due to difference in trial protocols. Additionally, causal effect if favipiravir in combination with dexamethasone in moderate to severe patients remain unexplored.

Therefore, this study aimed to evaluate the effectiveness of favipiravir on in-hospital mortality among patients with moderate to severe symptoms admitted to our field hospital setting. We compared patients who received favipiravir monotherapy or with additional systemic dexamethasone treatment to those who received symptomatic treatment based on standard care. Our main objective was to provide causal evidence of the treatments through an emulation of a target trial from observational data of the largest field hospital in Thailand.”

**3- Methods**

**Strengths:**
- The study design is robust, with careful consideration of biases such as immortal time bias.
- Comprehensive description of statistical techniques, including inverse probability of censoring weighting (IPCW) and restricted mean survival time (RMST) analysis.

**Criticisms:**
- The description of cloning and censoring might confuse readers unfamiliar with advanced epidemiological methods.
- Simplification or clarification is necessary.
- Potential residual confounding due to unmeasured variables (e.g., adherence to treatment protocols) is not addressed.

**Suggestions for Improvement:**
- Add a brief supplementary explanation or diagram illustrating the cloning-censoring process.
- Acknowledge the potential for residual confounding and discuss its impact on results.

**--------------------------------------------------------------**

**Response**

Thank you fir the suggestion in explanation imprivement. We have revised the study flow diagram in Figure 2 to imrove the demonstration of emulate target trial.

Furthermore, we have revised the discussion related to the methodology including the confounding as follows :

To compare survival outcome of treatments, the emulated target trial methodology is particularly relevant to our context because traditional observational analyses are prone to bias. First, imbalanced prognostic characteristics between treatment arms can result in a baseline confounding bias. Second, different treatment initiation times significantly reflect the immortal time bias [20, 21]. Patients who were assigned to received FPV and end up in the treated arm must survive until the treatment initiation. This artificially contributes to an inflated beneficial effect of FPV if both symptomatic treatment and FPV groups were equally defined at time zero and followed up while FPV groups must survive until prescription and the actual follow up begin. By emulating a trial protocol, we specifically defined a time zero of follow-up at admission for all treatment groups and allowed a grace period to receive FPV for one day after admission. Then, we cloned the patients to tripled and randomized them to each arm, thereby balancing the baseline across all study arms. The cloned data were censored, and the survival time analysis were weighted by censoring probability. Even the propensity score matching studies were also observed in favipiravir efficacy research [22-24], our study provides additional bias adjustment through the cloning, and censoring. From our analysis, the baseline BMI, duration from PCR to admission, underlying except DM and hypertension, and vaccination status were well balanced after weighting, while the remaining were later adjusted in the RMST model. Although the emulating a target trial may not fully replace an actual randomized controlled trial, but in a pandemic setting, this approach offers a rigorous way to extract causal insights from real-world data with minimized baseline imbalance and immortal time bias when RCTs are limited or delayed [25].

**4- Results**

**Strengths:**
- Results are well-organized with clear presentation of key findings through Kaplan-Meier curves and RMST analyses.
- Comprehensive demographic data provide context for interpreting the findings.

**Criticisms:**
- Baseline imbalances between treatment groups (e.g., age and vaccination status) may bias the findings, and while addressed statistically, their potential impact is not discussed adequately.
- The exclusion criteria (e.g., patients with delayed treatment initiation) could introduce selection bias, limiting generalizability.

**Suggestions for Improvement:**
- Discuss the extent to which baseline imbalances, even after weighting, could affect the study’s conclusions.
- Include sensitivity analyses or stratified results to explore the robustness of findings across subgroups (e.g., age or comorbidity status).

**--------------------------------------------------------------**

**Response เติม method 10% weighting**

Due to the emulation of target trial, we create clones for each arm before receiving the trial regimens. Therefore, at the baseline, the baseline of these three groups are equal. However, the weighting process helps improve the baseline as demonstrated in S2 table in supplementary files. We, additionally performed the adjustments in the primary outcome for factors that remain imbalance (STD >10%) after weighting [26]. In our case, after weighting, respiratory supports, gender, age, underlying diseases, and oxygen saturation remained imbalance (S1 Table).

**We have explained in statistical analysis section as follows :**

We used the standardized difference (STD) to assess the degree of differences in patient characteristics across the three groups (three comparison pairs). A significant difference between groups was defined as an absolute STD greater than 10% [26]. Any variables that remained imbalanced after weighting were adjusted in the weighted analysis model to ensure double robustness.

Moreover, after the primary analysis, we performed sensitivity analysis by excluding a group of less severe patients to specifically see favipiravir’s impact across subgruops (S2 Table).

**The resutls are explained in the text as follows:**

Furthermore, to explore robustness of the results, we performed sensitivity analysis among moderate-to-severe patients, patients with poor prognostic factors including hypoxia (oxygen saturation <96%, male gender, age more than 60 years, and without any vaccination were analyzed (S2 Table). As a result, the effect of favipiravir in reducing mortality was more pronounced among patients with moderate-to-severe (pneumonia and oxygen saturation <96%), patients with hypoxia, and male gender in either with or without dexamethasone.

**5- Discussion

Strengths:**
- The discussion places findings within the context of existing literature, effectively contrasting with other observational studies and randomized controlled trials.
- The limitations section is thorough, acknowledging the retrospective nature of the data and the early-pandemic context.

**Criticisms:**
- The discussion on favipiravir’s ineffectiveness could be expanded to consider potential mechanistic reasons for these findings, such as pharmacokinetic or pharmacodynamic limitations.
- While limitations are acknowledged, no actionable recommendations for future research are provided beyond the need for prospective trials.

**Suggestions for Improvement:**
- Add a brief discussion of potential pharmacological explanations for the lack of efficacy.
- Suggest specific future research directions, such as trials investigating favipiravir in specific subpopulations or combination therapies.
**--------------------------------------------------------------**

**Response**

We have revised the detail in discussion including phamacological explanation and implication as follows :

The observed effects of favipiravir and dexamethasone can be understood in light of their pharmacological actions. Favipiravir has broad activity against RNA viruses by inhibiting the viral RNA-dependent RNA polymerase of SARS-CoV-2 and was one of the first antivirals repurposed for COVID-19. By reducing viral load, favipiravir may shorten the period of high viral replication and potentially mitigate the direct cytopathic effects of the virus [27]. This results in a faster resolution of fever and pneumonia, and a lower likelihood that the infection triggers a severe inflammatory cascade [6, 28, 29]. Importantly, favipiravir’s efficacy seems more dependent on timing [15, 16]. In our moderate cases, favipiravir alone likely helped by curbing the virus during the acute phase, which is supported by the modest survival improvement and aligns with reports of quicker clinical improvement in other studies [28, 30]. In more severe cases, any antiviral effect of favipiravir would be adjunctive that a combination of favipiravir with other supportive therapy showed more favorable outcomes for COVID-19 patients [28]. This is biologically plausible because the ongoing viral load may continue in some patients driving inflammation and a markedly increase of inflammatory markers [31, 32]. This is where Dexamethasone plays a crucial role. Dexamethasone is a potent glucocorticoid that downregulates the production of pro-inflammatory cytokines, collectively reducing the inflammation-mediated lung injury that characterizes acute respiratory distress syndrome in severe COVID-19 [32-34]. This can be inferred that dexamethasone benefits the critically ill or hypoxic patients, [15, 35] but should not be used in mild cases who were not receiving respiratory support where possible harm can be observed due to impaired viral clearance from immunosuppression [15, 35].

With that being said, however, current variants and outpatient therapies have shifted the treatment landscape. Our study was conducted during the third wave in Thailand’s largest field hospital, where remdesivir was scarce and favipiravir served as the frontline antiviral. Though our results suggest modest benefit gains in survival time it is in the same range as that reported for remdesivir and other antiviral agents such as remdesivir Lopinavir/ ritonavir (S3 table) [23, 29, 36-38]. When used in conjunction with anti-inflammatory agents like dexamethasone, it may improve outcomes. Our findings suggest that favipiravir can be a valuable alternative in settings where other antivirals are inaccessible. It might not be the first choice for outpatients but could be useful for severe hospitalized patients with low oxygen saturation.

**6- Figures and Tables

Strengths:**
- Figures (e.g., Kaplan-Meier curves) and tables are clear and provide sufficient detail for readers to assess the results.

**Criticisms:**

- The font size in some figures is small, making them difficult to read.
- Captions are descriptive but could provide more context on the statistical methods applied.

**Suggestions for Improvement:**

- Improve figure readability by increasing font size and ensuring high resolution.
- Revise captions to briefly mention the key takeaways from the figures.

**--------------------------------------------------------------**

**Response**

We already revised the figure resolution as suggested. Moreover, the figure legen of figure 1-4 have revised as follows:

**Fig 1.** Study design using target trial emulation with cloning and censoring. A total of 3,193 eligible patients were initially assigned to one of three treatment groups: favipiravir + dexamethasone (FPV + Dexa), favipiravir (FPV) alone, or symptomatic treatment (ST). The cloning process expanded each patient across three treatment arms (n=9,579), ensuring comparability. Patients who deviated from their assigned protocol were censored, and follow-up was conducted for 30 days. Time-to-event analysis was performed using inverse probability censoring weights to estimate the treatment effects.

**Fig 2.** Exclusions of the participants. From all patients with COVID-19 atmittetd to Bussarakham Field hospital (n=18,184), asymptomatic patients (n=2,789) and mild severity (n=9,747) were excluded. The remaining eligible patients were 3,193 patients.

**Fig 3.** Standardize difference of each baseline character between treatment strategies (A) unweighted standardized difference; (B) weighted standardized difference. The reference line demonstrates STD at ±0.1, indicating the level of acceptable similarity between characters. Between ST and FPV with dexa, 13 baseline characters were different 9STD >0.1). Comparison between ST vs. FPV and FPV with dexa vs. FPV, were less different showing 10 and 9 out of 25 characters that were different, respectively.

**Fig 4.** Kaplan-Meier estimation of 30-day in-hospital mortality (right) and the predicted survival curve based on flexible parametric survival regression. The Kaplan-Meier curve shows empirical survival probabilities over 30 days for patients receiving symptomatic treatment (blue), favipiravir alone (red), and favipiravir combined with dexamethasone (green). The flexible parametric model provides a smoothed survival estimate, accounting for time-dependent effects. Both models indicate that survival probabilities differ across treatment groups, with the symptomatic treatment group showing a slightly lower survival probability over time.

**7- Ethical Considerations

Strengths:**

- The ethical approval process and waiver for informed consent are clearly described.
- Data availability is well-documented, adhering to open science principles.

**Criticisms:**

- The manuscript does not address potential ethical concerns regarding the use of data collected in emergency settings, such as the implications of waiving informed consent.

**Suggestions for Improvement:**

- Briefly discuss the ethical trade-offs of waiving consent in retrospective studies and the safeguards implemented to protect patient data.
**--------------------------------------------------------------**

**Response**

While retrospective studies inherently present ethical challenges regarding consent, the use of **anonymized data and compliance with ethical regulations** serves as a critical safeguard, allowing for the responsible use of real-world clinical data in research. Since this study was based on retrospective data collection, all treatments followed the Ministry of Public Health (MoPH) management guidelines and were administered solely at the discretion of the treating physicians, with no research-related interventions influencing clinical decisions. Because data were collected retrospectively from an existing database, obtaining informed consent from patients was not feasible.

To ensure patient confidentiality and data protection, all collected data were fully anonymized, excluding any personally identifiable information such as hospital numbers, identification numbers, names, or dates of birth. Furthermore, the study was conducted in compliance with institutional ethical guidelines for retrospective research, ensuring strict adherence to privacy and ethical standards.

**We addressed this to the ethical approval section as follows:**

The ethical approval was exempted by the Institutional Review Board and the Ethics Committee of Pranangklao Hospital (Approval ID: EC38/2021). There was no direct patient contact or primary collection of individual patient data. This study was based on retrospective data collection, all treatments followed the Ministry of Public Health (MoPH) management guidelines and were considered by the treating physicians, with no research-related interventions influencing clinical decisions. Because data were collected retrospectively from an existing database, obtaining informed consent from patients was not feasible. To ensure patient confidentiality and data protection, all collected data were fully anonymized excluding any personally identifiable information such as hospital numbers, identification numbers, names, or dates of birth.

**8- Conclusion

Strengths:**

- The conclusion effectively summarizes the findings and aligns them with the study’s objectives.

**Criticisms:**

- The conclusion does not sufficiently emphasize the study's implications for global COVID-19 treatment guidelines.
- Overemphasis on favipiravir’s lack of efficacy without suggesting contexts where it might still be useful.

**Suggestions for Improvement:**

- Reframe the conclusion to include a broader discussion of the findings’ relevance to current treatment paradigms and resource-limited settings.

Thank you

**--------------------------------------------------------------**

**Response**

We have revised the conclusion as follows :

**Conclusions**

Our emulated target trial suggests that favipiravir, especially when combined with dexamethasone, may offer modest survival benefits for hospitalized patients with moderate to severe COVID-19, particularly among patients with hypoxia. Our analysis contributes to the growing evidence in enhancing causal relationship of favipiravir in a real-world setting. While the benefits may not be substantial, favipiravir remains a practical oral antiviral in resource-limited environments. These findings support its use as an adjunct to corticosteroids when intervention is feasible, although further controlled studies are needed to strengthen clinical recommendations.

**Reviewer #2**:

 I would like to express my gratitude to the editors for providing me with the opportunity to review this intriguing manuscript.

**Comment 1:**
The manuscript does not describe whether all the assumptions for target trial emulation (consistency, positivity, and exchangeability) were explicitly tested or validated.
The creation of clones for patients assumes that treatment assignment does not influence subsequent outcomes—a critical assumption that may not be fully met in real-world settings.
**--------------------------------------------------------------**

**Response**

We acknowledge the importance of validating the key assumptions of target trial emulation (TTE): exchangeability, consistency, and positivity. Below, we clarify how these assumptions were addressed in our study.

Exchangeability assumes that treatment groups are comparable and that there is no unmeasured confounding. While this assumption is inherently unverifiable, we believe that we have adjusted for a sufficient set of confounders in our analysis and mitigated further selection bias during follow-up by employing inverse probability of censoring weighting.

Consistency requires that each treatment level corresponds to a well-defined intervention, and that the treatment effect is not influenced by variations in how the intervention is administered. In our study, treatments were clearly defined and recorded in the patient database according to standardized hospital protocols. This ensured that each treatment level corresponded to actual clinical practice, thereby preserving consistency.

Positivity requires that all patients have a non-zero probability of receiving any treatment option. In our analysis, we performed weight truncation at both extremes to ensure that the weights were stabilized and that the positivity assumption was upheld.

**We added the followings to the methods section:** The censoring weights were calculated as the inverse of the predicted probabilities, estimated separately for each treatment group. Weights were truncated at the 99th percentile to remove extreme values, thereby mitigating the risk of violating the positivity assumption.

**We added the followings within the limitation of the manuscript:** The small sample size in the symptomatic treatment group may have reduced statistical power, introducing instability and variability in treatment effect estimates, and potentially affecting the assumptions of exchangeability and positivity. To address this, we employed inverse probability of censoring weighting (IPCW), adjusted for imbalance factors, and conducted sensitivity analyses. However, limited sample size may still challenge the validity of these assumptions. Additionally, despite adjusting for a comprehensive set of covariates, residual unmeasured confounding cannot be excluded, which may compromise exchangeability.

**Comment 2**
The description of how censoring weights were calculated is not detailed. Lack of transparency about how weights were derived can affect reproducibility and credibility of results
**--------------------------------------------------------------**

**Response revise model ใหม่**

We used cox regression to estimate probability for being censored. The weight model include age more than 60, gender, time from PCR to admit, respiratory supports, cerebrovascular, cardiovascular diseases, cirrhosis, chronic kidney diseases, chronic obstructive pulmonary diseases, diabetes, hypertension, immunodeficiency, obesity body temperature, heart rate, BMI sistolic and diastolic blood pressure, oxygen saturation, and vaccine status.

**We revised the statistical analysis section as follows:**

Based on clinical knowledge, the Cox model included all potential prognostic factors and confounders. The weight model include age more than 60, gender, time from PCR to admit, respiratory supports, cerebrovascular, cardiovascular diseases, cirrhosis, chronic kidney diseases, chronic obstructive pulmonary diseases, diabetes, hypertension, immunodeficiency, obesity body temperature, heart rate, BMI sistolic and diastolic blood pressure, oxygen saturation, and vaccine status. The censoring weights were subsequently determined by inverting the predicted probability, which was calculated separately for each treatment plan.”

**Comment 3:**
The manuscript identifies moderate imbalances in baseline characteristics across treatment groups, even after weighting (Table S1), including variables such as age, comorbidities, and initial respiratory support. These imbalances may undermine the validity of causal inferences and could necessitate sensitivity analyses to adjust for these covariates and reduce bias.
Additionally, key factors such as the WHO ordinal scale, hypoxemia on admission, and the duration from symptom onset to hospital admission should also be accounted for in the analysis. Importantly, the duration from PCR testing to admission is not a valid measure and does not adequately represent the likelihood of increased disease severity.

**--------------------------------------------------------------**

**Response**

To address this concern, the model for primary analysis of restricted mean survival time was adjusted using the parameters that remain imbalance after weighting. These include **age, gender, duration from PCR test date to admission, BMI, comorbidities, oxygen saturation (%), blood pressure, respiratory supports**. Furthermore, we conducted sensitivity analysis based on imbalance characters including, age more than 60, gender, and hypoxia. The results demonstrated consistent trend to the primary analysis where the symptomatic treatment was significantly inferior to the others, especially among hypoxia patients.

Furthermore, the evolving management strategies during the pandemic also introduced variability in the exact time from symptom onset to hospital admission, as patients' decision to seek medical attention was influenced by changing guidelines and resource availability. To address this, we replaced the time from symptom onset to admission with the “PCR test date to admission”, as this variable was more reliable based on field hospital and pandemic situations, widely available across all admitted patients, and free from recall bias. In addition, the WHO ordinal scale was not solidified and implemented at our field hospital leading to missing data on this variable. However, since all patients in this study were hospitalized, the WHO severity scale would range from 4 to 10. Therefore, it would be best to acknowledge this to our limitations.

**We mentioned this in the limitation section as follows:**

Third, due to the retrospective nature of our study and the field hospital setting, the completeness of certain baseline characteristics was inherently limited. This includes data on the WHO COVID-19 ordinal scale, CRP levels, anti-interleukin treatment, disease progression, progression to mechanical ventilation, and time to discharge. Although we have respiratory support data, we lack details on the vasopressor and ECMO. However, since all patients in this study were hospitalized, the WHO severity scale would range from 4 to 10. Fourth, the management strategies during the pandemic also introduced variability in the exact time from symptom onset to hospital admission. We therefore replaced the time from symptom onset to admission with the PCR test date to admission, as this variable was more reliable, widely available across all admitted patients, and free from recall bias.

**Comment 4:**

The absence of key baseline characteristics, such as obesity or morbid obesity (defined as BMI >30 kg/m², as opposed to using body weight >90 kg, which is insufficient—please review the proper definition of obesity), the time from symptom onset to hospital admission, the WHO COVID-19 severity scale, baseline hypoxemia, and the precise duration from symptom onset to hospitalization (e.g., distinguishing between 3 days and 7 days, as these may have significantly different implications) raises concerns about potential bias and the validity of the study. Furthermore, the lack of data on CRP levels, the percentage of pulmonary involvement on chest X-ray, and progression to the need for mechanical ventilation further limits the robustness of the findings and may compromise the reliability of the conclusions drawn.

**--------------------------------------------------------------**

**Response**

We acknowledge the reviewer's concerns regarding the baseline characteristics and have revised the obesity criteria according to the Bussarakham Field Hospital treatment guideline, where BMI >35 kg/m² or body weight >90 kg was considered obesity. This adjustment complied with the hospital’s treatment protocols and risk group classification.

Additionally, due to the retrospective nature of our study and the field hospital setting, the completeness of certain baseline characteristics was inherently limited. This includes data on the WHO COVID-19 ordinal scale, CRP levels, disease progression, progression to mechanical ventilation, and time to discharge. Although we have respiratory support data, we lack details on the vasopressor and ECMO. However, since all patients in this study were hospitalized, the WHO severity scale would range from 4 to 10.

Furthermore, the evolving management strategies during the pandemic also introduced variability in the exact time from symptom onset to hospital admission, as patients' decision to seek medical attention was influenced by changing guidelines and resource availability. To address this, we replaced the time from symptom onset to admission with the “PCR test date to admission”, as this variable was more reliable based on field hospital and pandemic situations, widely available across all admitted patients, and free from recall bias. In addition, the WHO ordinal scale was not solidified and implemented at our field hospital leading to missing data on this variable. However, since all patients in this study were hospitalized, the WHO severity scale would range from 4 to 10. Therefore, it would be best to acknowledge this to our limitations.

**We mentioned this in the limitation section as follows:**

Third, due to the retrospective nature of our study and the field hospital setting, the completeness of certain baseline characteristics was inherently limited. This includes data on the WHO COVID-19 ordinal scale, CRP levels, anti-interleukin treatment, disease progression, progression to mechanical ventilation, and time to discharge. Although we have respiratory support data, we lack details on the vasopressor and ECMO. However, since all patients in this study were hospitalized, the WHO severity scale would range from 4 to 10. Fourth, the management strategies during the pandemic also introduced variability in the exact time from symptom onset to hospital admission. We therefore replaced the time from symptom onset to admission with the PCR test date to admission, as this variable was more reliable, widely available across all admitted patients, and free from recall bias.

**Comment 5:**
The authors did not differentiate between moderate and severe COVID-19 cases, which is a critical oversight. For moderate COVID-19, outcomes such as disease progression and hospital admission are more appropriate measures, rather than mortality. Conversely, severe COVID-19 should be evaluated using metrics such as the need for mechanical ventilation, ICU admission or transfer to a nearby tertiary hospital, ICU length of stay, development of ARDS, respiratory failure, and mortality.
This lack of distinction may stem from an inappropriate study design, which fails to account for the differing clinical trajectories and outcome measures relevant to moderate and severe cases.
**--------------------------------------------------------------**

**Response**

We acknowledge the reviewer's concern regarding the distinction between **moderate and severe COVID-19 cases** and the appropriate outcome measures for each group. However, due to unavailability from retrospective field hospital’s data collection, we were unable to systematically assess disease progression, time from admit to refer, need of mechanical ventilation, or respiratory failure outcomes. We emphasize the critical endpoint of disease, an in-hospital mortality, which remains a major concern even after ICU admission.

Additionally, ICU admission and ICU length of stay were observed in only 18 patients who received favipiravir and dexamethasone. This small number of event limits the ability to draw meaningful conclusions.

**This is acknowledged as a key limitation of our study, and we have included this issue in the discussion section as follows:**

Our study focusses mainly on morality rather than disease progression or ICU admission rate which are commonly used to explain disease trajectory in moderate COVID-19 cases. Instead, we emphasize the critical endpoint of disease, an in-hospital mortality, which remains a major concern even after ICU admission. Future studies with more comprehensive clinical outcomes will be necessary to better assess treatment impact across different severity levels.

**Comment 6**
The model used to predict the probability of censoring includes "all potential prognostic factors and confounders," but the manuscript lacks justification for the variables included and excluded. These were the key confounders that led to compromise results.
**--------------------------------------------------------------**

**Response**

We used cox regression to estimate probability for being censored. The weight model include age more than 60, gender, time from pcr to admit, respiratory supports, cerebrovascular, cardiovascular diseases, cirrhosis, chronic kidney diseases, chronic obstructive pulmonary diseases, diabetes, hypertension, immunodef, obesity body temperature, heart rate, BMI, sistolic and diastolic blood pressure, oxygen saturation, and vaccine status.

**We revised the statistical analysis section as follows:**

“	Based on clinical knowledge, the Cox model included all potential prognostic factors and confounders. The weight model include age more than 60, gender, time from pcr to admit, respiratory supports, cerebrovascular, cardiovascular diseases, cirrhosis, chronic kidney diseases, chronic obstructive pulmonary diseases, diabetes, hypertension, immunodef, obesity body temperature, heart rate, BMI sistolic and diastolic blood pressure, oxygen saturation, and vaccine status. The censoring weights were subsequently determined by inverting the predicted probability, which was calculated separately for each treatment plan.”

**Comment 7**
Restricted Mean Survival Time differences are reported, the manuscript does not clearly indicate if assumptions for parametric survival models were met or if alternative models were considered.
**--------------------------------------------------------------**

**Response**

We follows the recommended protocol illustrated by Maringa et al., 2020. The Restricted Mean Survival Time (RMST) provides a robust alternative for comparing survival outcomes when PH assumptions do not hold. Unlike Cox models, RMST does not rely on the proportionality of hazards, making it more suitable for real-world clinical data with varying treatment effects over time. Our approach aligns with established recommendations for survival analysis in such scenarios [39]. Additionally, we considered alternative models; however, RMST was chosen for its ability to provide an intuitive and clinically interpretable measure of survival differences across treatment groups.

**We addressed this issue in the statistical analysis section as follows:**

The restricted mean survival time (RMST) difference was employed to evaluate the mean survival times between the three groups for the primary study endpoint, in-hospital death. The RMST provides a robust alternative for comparing survival outcomes when PH assumption is violated. Unlike Cox models, RMST does not rely on the proportionality of hazards, making it more suitable for real-world clinical data with varying treatment effects over time [39].

**Comment 8**
The very small sample size in the symptomatic treatment group may result from inadequate statistical power. A limited sample size reduces the reliability of estimated effects because statistical power depends on the number of actual observed events rather than an artificially balanced dataset. This limitation likely contributed to the low number of observed events in the standard treatment group.
Moreover, the cloning approach, which creates clones for all treatment groups, has inherent challenges. Clones assigned to groups that do not align with the original treatment are censored early, leading to shorter follow-up times and fewer data points available to estimate treatment effects. This issue is particularly pronounced for smaller sample sizes, as it results in unstable or extreme weights for clones in the symptomatic treatment group. Such instability inflates variance in the analysis and further reduces the statistical power, undermining the robustness of the findings.
As a result, the symptomatic treatment group may lack sufficient representation for a robust and reliable comparison. To address this, more sophisticated weighting methods should be employed to improve stability and mitigate the issues associated with extreme weights. Additionally, a thorough sensitivity analysis should be conducted to assess the robustness of the results and ensure that the findings are not unduly influenced by the limitations of the current approach.
**--------------------------------------------------------------**

**Response**

We acknowledge the concern regarding the small sample size in the symptomatic treatment group and its potential impact on statistical power. This issue is addressed in the limitations section, where we note that sample size imbalance across groups, particularly in the symptomatic treatment group, may introduce instability and variability in treatment effect estimates.

To mitigate this, we employed weight and adjustment methods using inverse probability consoring weight. After weighting, factors with standard difference >10% were further adjusted in the model to enhance the robustness. However, we recognize that cloning methods, which assign patients to multiple treatment groups, may lead to early censoring of clones that do not align with their treatment protocol. This could shorten follow-up times and reduce the number of data points available for estimating treatment effects, particularly in smaller groups, such as the symptomatic treatment cohort.

To address concerns about instability due to extreme weights, we performed a sensitivity analysis focusing on key imbalanced characteristics, including age >60, gender, and hypoxia (oxygen saturation <96%). The results remained consistent with our primary analysis, demonstrating that symptomatic treatment was significantly inferior to the other treatment groups, particularly among hypoxic patients.

Additionally, we revised our analysis by separating more severe cases from the moderate group to improve treatment comparisons. This refinement resulted in more pronounced differences, with the antiviral + dexamethasone group showing a greater RMST difference compared to symptomatic treatment (2.83; 95% CI: 0.81, 4.86; p = 0.006), and the FPV-alone group also demonstrating a significant RMST difference compare to symptomatic group (2.54; 95% CI: 0.10, 4.98; p = 0.041). These findings are presented in S2 Table.

**We added this concerns in the limitation section as folows:**

“In addition, the small sample size in the symptomatic treatment group and its potential impact on statistical power may introduce instability and variability in treatment effect estimates. To mitigate this, we employ the IPCW with further the adjustment of imbalance factors to the model, and the sensitivity analysis that could help ensure the robustness of the results.”

**Comment 9**
The in-hospital mortality data should be further classified to enhance clarity. I recommend that the authors analyze 7, 14-day and 30-day mortality rates to provide a more detailed perspective and facilitate a clearer discussion of the survival analysis.
**--------------------------------------------------------------**

**Response**

In our study population, the overall number of mortality events was low (n=32). Conducting a survival analysis over narrower time frames, such as 7-day or 14-day mortality, would result in even fewer observed events, potentially compromising statistical power and leading to unstable estimates. From our dataset, only 13 deaths occurred within the first 7 days, while 28 deaths occurred within 14 days.

However, we acknowledge the value of further stratification and have included this as a limitation and consideration for future research. We suggest that larger studies with higher statistical power should explore 7-day and 14-day mortality rates to provide more granular insights into early survival outcomes.

**We addressed this issue in the limitation section as follows:**

Further studies, with larger sample size, higher statistical power should explore 7-day and 14-day mortality rates to provide clearer insights into early survival outcomes. Lastly, our results, derived from an uncontrolled real-world setting, should be validated by prospective controlled trials to confirm their applicability.

**Comment 10:**
The handle with missing data should be mentioned.
**--------------------------------------------------------------**

**Response**

We use imputation method to handle missing data using the kNN imputation. Data excluding classification factors (age, gender, symptomatic status, favipiravir treatment status, dexamethasone treatment status) were included in imputation procedures.

**The process was demonstrated in the methodology section as follows:**

**Missing data handling**

Missing values in prognostic factors were addressed through imputation using the k-Nearest Neighbor (kNN) imputation based on a variation of the Gower Distance [40]. After excluding classification factors (age, gender, symptomatic status, favipiravir treatment status, dexamethasone treatment status), only prognostic factors were included in imputation procedures, while outcome variables (time and mortality status) were excluded. The imputation was conducted in R using a kNN function from VIM package with default arguments setting at 5 neighbors, median function for numerical data, and maximum function for categorical data. The imputed variables were then merged with outcome variables, and the data were used for model derivation.

**Comment 11:**
The authors should address the generalizability of their findings, given that the study was conducted within a specific context—a large field hospital in Thailand—during a particular wave of the COVID-19 pandemic. This unique setting and time frame may limit the applicability of the results to other populations, healthcare settings, or pandemic phases.
**--------------------------------------------------------------**

**Response**

We acknowledge that this setting may differ from those in later phases of the pandemic, especially where vaccination rates increased, viral variants evolved, and access to newer antivirals improved. Indeed, our study was conducted within a specific context—a large field hospital in Thailand—during the third wave of the COVID-19 pandemic [7], when resources were constrained, remdesivir was not widely available, and favipiravir was used as the frontline antiviral. These conditions shaped both clinical practices and treatment accessibility.

However, we believe our findings remain informative in several ways. First, the use of an emulated target trial framework with robust adjustment for confounding and immortal time bias enhances the internal validity and causal interpretability of our results. Second, the modest but consistent survival benefit observed with favipiravir, particularly among patients with hypoxia, is aligned with findings from several other studies conducted in diverse settings.

**We have now added a paragraph in the discussion as follows:**

With that being said, however, current variants and outpatient therapies have shifted the treatment landscape Our study was conducted in the unique context of Thailand’s largest field hospital during the country’s third wave of the COVID-19 pandemic (May–September 2020), when the access to remdesivir was limited and favipiravir was used as the frontline antiviral [7]. At that time, the Thai government primarily imported favipiravir, and secure our own favipiravir supply, producing generic favipiravir tablets domestically. Therefore, favipiravir became the most widely used antiviral for symptomatic COVID-19 patients with risk factors [41]. However, although available, remdesivir was reserved for select cases based on physician discretion, particularly for pregnant women in their first trimester to avoid teratogenic effects and patients unable to take oral medications due to absorption issues. This context reflects a setting where treatment decisions influenced by medication availability and evolving clinical practices. While these circumstances may differ from later phases of the pandemic, particularly with the emergence of new variants, expanded vaccination coverage, and broader access to alternative antivirals, they represent real-world conditions faced by constraint setting during critical surges. These setting-specific factors may limit the generalizability of our findings to other populations or healthcare systems. However, the use of an emulated target trial framework in our analysis enhances the internal validity and supports causal inference. Additionally, the observed survival benefit of favipiravir, particularly in hypoxic patients, is consistent with findings from other trials and observational studies. Though our results suggest modest benefit gains in survival time of favipiravir, it is in the same range as that reported for remdesivir and other antiviral agents such as remdesivir Lopinavir/ ritonavir (S3 table) [23, 29, 36-38]. When used in conjunction with anti-inflammatory agents like dexamethasone, it may improve outcomes. Our findings suggest that favipiravir can be a valuable alternative in settings where other antivirals are inaccessible. It might not be the first choice for outpatients but could be useful for severe hospitalized patients with low oxygen saturation.

**Comment 12:**
The use of anti-inflammatory treatments, particularly anti-IL-6 therapies or the specific dose of dexamethasone, should be clearly detailed in the baseline characteristics, methods, and subgroup analysis
**--------------------------------------------------------------**

**Response**

This is our major limitation that we cannot find data on anti interleukin treatments. However, the dosage of dexamethasone is provided in methodology section as indicated by the Thai MoPH guideline.

**We addressed this in the limitation section as follows:**

Third, due to the retrospective nature of our study and the field hospital setting, the completeness of certain baseline characteristics was inherently limited. This includes data on the WHO COVID-19 severity scale, CRP levels, anti-interleukin treatment, disease progression, progression to mechanical ventilation, and time to discharge. Although we have respiratory support data, we lack details on the vasopressor and ECMO. However, since all patients in this study were hospitalized, the WHO ordinal scale would range from 4 to 10.

**Moreover, the dosage and treatment strategies of dexamrthasone was also addressed as follows:**

The MoPH recommends a favipiravir dose of 1800 mg twice daily (BID) on day 1 and 800 mg BID on the next days (5-14 days) for COVID-19, and dexamethasone oral dose of 20 mg loading at first dose, then 5 mg daily for 5-7 days. Patients weighing over 90 kg received an increased FPV dose, specifically Day 1 for 12 tablets (200 mg) twice a day, followed by Day 2-5 for 5 tablets (200 mg) twice a day. For pneumonia with SpO2 ≤ 94% or a rapid symptom increase, dexamethasone 6 mg/day for 7-10 days is recommended, with dose adjustments for weight over 90 kg. If SpO2 ≤ 93% or oxygen ≥ 3 L/min is required, dexamethasone up to 20 mg/day can be given, tapering as symptoms improve over at least 7 days. For severe cases requiring High-Flow Nasal Cannula, Non-Invasive Ventilation, or mechanical ventilation, dexamethasone 20 mg/day should be administered for at least 5 days, followed by gradual tapering. If symptoms worsen, increasing the dose should be carefully considered against the risk of infections.

**Comment 13:**
A subgroup survival analysis stratified by moderate and severe COVID-19 cases is essential to clarify the distinct outcomes in these groups and to enhance the generalizability of the findings
**--------------------------------------------------------------**

**Response**

Thank you for the concern in moderate and severe cases.

The majority of populations are moderate cases. We revised the analysis as suggested by performing the sensitivity analysis among patients with severe illness and excluding the moderate cases. As a result, the groups receiving anti-viral agent and dexamethasone provided a slightly more pronounced resutls that the group receiving FPV alone when compared to symtomatic treatment with RMST difference 2.83 (95%CI 0.81,4.86; p-value = 0.006) and 2.54 (95%CI 0.10,4.98; p-value =0.041). The resutls are illustrated in **S3 Table**.

**Comment 14:**
Line 95-102: More literature should be reviewed about the outcome of favipiravir in each severity, which was needed in the introduction. Many global reports have been published with different severity, including in Thailand, that the authors should provide citations.
**--------------------------------------------------------------**

**Response**

**Thank you for the sugestions. We elaborated more on the literature in the introduction section as follows:**

Despite its widespread use, favipiravir’s clinical efficacy in COVID-19 remains controversial [1, 10]. Several clinical trials focused primarily on mild COVID-19 cases, where favipiravir's effect is minimal, as most patients naturally recover with supportive care [11, 12]. However, in moderate to severe patients who are facing higher risks of respiratory failure and complications, antiviral and anti-inflammatory therapies may play a crucial role in improving outcomes [13, 14]. In addition, the study from RECOVERY trial demonstrated that dexamethasone, a potent anti-inflammatory steroid, reduced 28-day mortality in COVID-19 patients but not among those receiving no respiratory support [15]. Therefore, during the pandemic wave, the Thai MoPH guideline suggested the use of favipiravir in combination dexamethasone in patients with moderate to severe symptom.

In Thailand, studies investigated the use of favipiravir in COVID-19 patients are partly limited. Studies by Sirijatuphat et al. [16] and Siripongboonsitti et al. [17] compare efficacy of favipiravir to control treatment and found that favipiravir showed improvements on clinical outcomes in mild to moderate COVID-10 patients. Rattanaumpawan et al. [18] reported the favipiravir’s effectiveness in reducing hospital stays among hospitalized patients, though its impact on mortality remained unclear. Furthermore, a real-world study during the Delta variant wave indicated that favipiravir monotherapy reduced the 28-day mortality risk in severe COVID-19 (Relative risk = 0.72 (95% CI 0.58–0.91; P = 0.006) but not in mild and moderate patients [19]. The results are still inconclusive due to difference in trial protocols. Additionally, causal effect if favipiravir in combination with dexamethasone in moderate to severe patients remain unexplored.

**Comment 15:**
Line 138-145: To facilitate easier understanding for the audience, the authors should provide a clear description of the phase of the COVID-19 outbreak during the study period in the specific setting.
**--------------------------------------------------------------**

**Response**

According to the anouncement of MoPH of Thailand, the period was in the phase 3, which started at 1 April 2021.

**We provide this information to the introduction section as follows:**

In Thailand, as in other countries, favipiravir was rapidly adopted as a first-line antiviral treatment for COVID-19 following its emergency authorization in 2020. [3-6]. This drug was included in the Thai Ministry of Public Health (MoPH) guidelines and widely used during the peak of the pandemic, particularly in response to the third phase that was driven by the delta variant in 2021 [7, 42].

**Comment 16:**
I have come across several studies that explore increasing the dose of favipiravir (FPV) for obese patients. However, this important aspect is not mentioned or addressed in the current study.
**--------------------------------------------------------------**

**Response**

We acknowledge the reviewer's concern regarding the dosing of favipiravir (FPV) in obese patients. In our study, dose adjustments for obese patients followed the treatment guidelines used at Bussarakham Field Hospital. Patients weighing over 90 kg received an increased FPV dose, specifically Day 1 for 12 tablets (200 mg) twice a day, followed by Day 2-5 for 5 tablets (200 mg) twice a day.

**We revised the treatment strategies in methodology section as follows:**

**Treatment Strategies and Assignments**

The MoPH recommends a favipiravir dose of 1800 mg twice daily (BID) on day 1 and 800 mg BID on the next days (5-14 days) for COVID-19, and dexamethasone oral dose of 20 mg loading at first dose, then 5 mg daily for 5-7 days. Patients weighing over 90 kg received an increased FPV dose, specifically Day 1 for 12 tablets (200 mg) twice a day, followed by Day 2-5 for 5 tablets (200 mg) twice a day. For pneumonia with SpO2 ≤ 94% or a rapid symptom increase, dexamethasone 6 mg/day for 7-10 days is recommended, with dose adjustments for weight over 90 kg. If SpO2 ≤ 93% or oxygen ≥ 3 L/min is required, dexamethasone up to 20 mg/day can be given, tapering as symptoms improve over at least 7 days. For severe cases requiring High-Flow Nasal Cannula, Non-Invasive Ventilation, or mechanical ventilation, dexamethasone 20 mg/day should be administered for at least 5 days, followed by gradual tapering. If symptoms worsen, increasing the dose should be carefully considered against the risk of infections.

**Comment 17**
The authors should discuss why severe COVID-19 cases in the study did not receive remdesivir as the antiviral treatment, particularly given its established role in managing severe cases. To the best of my knowledge, favipiravir has not been approved for use in severe COVID-19 cases since the onset of the pandemic. Addressing this issue would clarify the rationale behind the choice of antiviral treatment and provide a more comprehensive context for interpreting the study’s findings.
**--------------------------------------------------------------**

**Response**

During Thailand’s third wave of the pandemic, favipiravir was the most accessible antiviral and was widely prescribed, both alone and in combination with dexamethasone. At that time, the Thai government primarily imported favipiravir, which became the most widely used antiviral for symptomatic COVID-19 patients with risk factors. However, although available, remdesivir was reserved for select cases based on physician discretion, particularly for pregnant women in their first trimester (to avoid teratogenic effects) and patients unable to take oral medications due to absorption issues. These patients were either given remdesivir alone or favipiravir alone, but combination therapy was not standard practice.

As the pandemic escalated, Thailand took measures to secure our own favipiravir supply, with the Government Pharmaceutical Organization (GPO) producing generic favipiravir tablets domestically [41]. In July 2021, the Thai FDA formally approved the locally manufactured version, which was about half the cost of imported favipiravir, making it the predominant antiviral used during the third wave. Since then, favipiravir has been widely available under emergency use or off-label use, with millions of doses distributed through government healthcare programs.

**Comment 18**
The definition provided for a moderate COVID-19 case, “The moderate case is a symptomatic patient with at least one risk factor and the need for respiratory support,” is incorrect and requires significant revision. A proper definition, supported by appropriate citations, should be included. For example, according to the WHO clinical management guidelines, moderate COVID-19 is typically defined as a symptomatic patient with evidence of lower respiratory tract infection (e.g., clinical signs of pneumonia) but without the need for supplemental oxygen. The authors should revise this statement to align with established guidelines and provide a clear reference..
**--------------------------------------------------------------**

**Response**

In this study, we categorized COVID-19 severity based on Thailand’s national guidelines on clinical practice, diagnosis, treatment, and prevention of healthcare-associated infections for COVID-19. A moderate case was defined as a patient with mild pneumonia or symptomatic illness with at least one risk factor predisposing them to severe disease progression. This classification aligns with the Ministry of Public Health (MoPH) guidelines and it determined how the patients received the treatments [4].

However, to align with the **WHO clinical management guidelines and for generalizability**, we revised the classification as follows:

**Old version :**

- Mild case is a symptomatic patient without risk factors for severe condition and received no respiratory support

- Moderate case is a symptomatic patient with at least one risk factor and the need for respiratory support, as well as a patient who has COVID-19 pneumonia disease but has an oxygen saturation >96%

- Severe case is a patient with pneumonia with oxygen saturation ≤96%.

**Revised version:**

- Mild case is symptomatic patient without evidence of viral pneumonia or hypoxia

- **Moderate case** is a symptomatic patient with **pneumonia** or symptomatic patient with **one or more risk factors severe COVID-19 (including**.

**- Moderate-to-severe case is** symptomatic patients with **pneumonia and oxygen saturation ≤96%**.

**We revised the eligibility section in methodology as follows:**
Patients with risk factors for severe COVID-19 include those who aged ≥60 years old, or obesity (BMI >35 kg/m^2^ or body weight ≥ 90 kg), or with at least one comorbidity. Disease severity was categorized according to the **WHO clinical management and Thai MoPH guidelines [4, 43]**: a mild case is symptomatic patients without evidence of viral pneumonia or hypoxia; a moderate case a symptomatic patient with at least one risk factor and the need for respiratory support, as well as a patient who has COVID-19 pneumonia disease but has an oxygen saturation >96%; a moderate-to-severe case is symptomatic patients with pneumonia and oxygen saturation ≤96%. This is moderate-to-severe case was not defined in the WHO guideline and they are generally categorized as moderate case; however, we specifically defined this group based on our MoPH and clinical management during the pandemic [4].

**Comment 19**
The authors should provide an explanation for the variation in treatment regimens among patients, specifically why some received favipiravir (FPV) alone, others received a combination of FPV and dexamethasone, and why certain patients were managed with only symptomatic treatment. Clarifying these treatment decisions, including the clinical rationale, patient characteristics, or resource availability that influenced them, is essential to understanding the study design and its implications..
**--------------------------------------------------------------**

**Response**

The variation in treatment regimens among patients in our study was guided by clinical severity, patient risk factors, and oxygen requirements, and clinician’s consideration as outlined in the **doctor order from Bussarakham Field Hospital** (S1 Figure). These treatment decisions were influenced not only by clinical severity and risk stratification but also by resource availability and evolving treatment guidelines during the pandemic. The use of dexamethasone was based on clinician’s considerations as follows:

“• If pneumonia is present and SpO2 ≤ 94% or if SpO2 during exertion decreases by ≥ 3% from the initial measured value, or if the patient shows a rapid increase in symptoms, consideration may be given to administering this when SpO2 ≤ 96% on a case-by-case basis. Administer dexamethasone 6 mg/day for 7-10 days. If the weight is over 90 kg, consider adjusting the dose upward.

• If there is pneumonia with SpO2 ≤ 93% or if oxygen supplementation is required at ≥ 3 L/min, consider administering dexamethasone up to 20 mg/day or equivalent. Adjust the dose downward if symptoms improve, with a total treatment duration of at least 7 days.

• If there is pneumonia requiring HFNC (High-Flow Nasal Cannula), NIV (Non-Invasive Ventilation), or mechanical ventilation, administer dexamethasone 20 mg/day for at least 5 days, then gradually taper the dosage as the patient's symptoms improve. If symptoms worsen, increase the dosage after weighing the benefits against the risks of superimposed infections.”

However, treatment was based on consideration and expertise / background. therefore, variation in receiving treatment was observed.

**We added this information to the methodology section as follows:**

The MoPH recommends a favipiravir dose of 1800 mg twice daily (BID) on day 1 and 800 mg BID on the next days (5-14 days) for COVID-19, and dexamethasone oral dose of 20 mg loading at first dose, then 5 mg daily for 5-7 days. Patients weighing over 90 kg received an increased FPV dose, specifically Day 1 for 12 tablets (200 mg) twice a day, followed by Day 2-5 for 5 tablets (200 mg) twice a day. For pneumonia with SpO2 ≤ 94% or a rapid symptom increase, dexamethasone 6 mg/day for 7-10 days is recommended, with dose adjustments for weight over 90 kg. If SpO2 ≤ 93% or oxygen ≥ 3 L/min is required, dexamethasone up to 20 mg/day can be given, tapering as symptoms improve over at least 7 days. For severe cases requiring High-Flow Nasal Cannula, Non-Invasive Ventilation, or mechanical ventilation, dexamethasone 20 mg/day should be administered for at least 5 days, followed by gradual tapering. If symptoms worsen, increasing the dose should be carefully considered against the risk of infections.

**Comment 20:**
In Table 2, it is evident that the FPV-alone and symptomatic treatment groups included a higher proportion of patients without hypoxemia, indicating that the majority of moderate COVID-19 cases in these groups were less severe. Conversely, more than 50% of patients in the FPV/dexamethasone group required oxygen supplementation, compared to less than 18% in both the FPV-alone and symptomatic treatment groups. This disparity suggests that the FPV-alone and symptomatic treatment groups had lower disease severity, which could introduce bias when evaluating the treatment effectiveness across these groups.
**--------------------------------------------------------------**

**Response**

Thank you for conern about bias in FPV-alone and symptomatic treatment groups when evaluating the treatment effectiveness across these groups. However, we acknowledge that this study is an emulation of target trial. We therefore use the weighting with an effort to balance the baseline. We address this concern by doing the sensitivity analysis (S3 Table).

The results showed that, among patients with hypoxia (oxygen saturation <96%), the results are consistent with the primary analysis. Specifically, both anti-viral groups have significantly higher RMST (p-value 0.020 and 0.036).

**Comment 21**
Please check the standard abbreviations in Table 2
**--------------------------------------------------------------**

**Response**

All abbreviations in table 2 were chacked and revised.

**Comment 22**
Line 365-373: These statements were invalid. While dexamethasone has demonstrated clear benefits in severe COVID-19 cases, the data in the study indicate that 44% of patients in the dexamethasone group had non-severe COVID-19. This means that only 56% of patients with severe COVID-19 might have benefitted from dexamethasone, raising questions about its appropriate use in this cohort.
This reinforces my recommendation for the authors to analyze moderate COVID-19 cases separately from severe COVID-19 cases. Evaluating severe COVID-19 in isolation may downplay the role of antiviral agents, as these cases are often beyond the viral replication phase where antiviral drugs are most effective. In contrast, moderate COVID-19 cases, which are typically in the viral replication phase, could demonstrate a more pronounced benefit from antiviral treatments.
**--------------------------------------------------------------**

**Response**

Thank you for the concern in moderate and severe cases. We revised the analysis as we separate the more severe cases from the moderate group. As a result, the groups receiving anti-viral agent and dexamethasone provided a slightly more pronounced resutls that the group receiving FPV alone when compared to symtomatic treatment with RMST difference 2.83 (95%CI 0.81,4.86; p-value = 0.006) and 2.54 (95%CI 0.10,4.98; p-value =0.041). The resutls are illustrated in **S2 Table.**

**Comment 23:**
I recommend that the authors engage in a more in-depth discussion to highlight the novelty of their research for the audience. This should include a detailed explanation of the authors' specific methodology, emphasizing how it differs from or improves upon existing approaches
**--------------------------------------------------------------**

**Response**

**We have revised the discussion as follows:**

Our study aimed to assess the efficacy of favipiravir in moderate to severe COVID-19 patients by using observational data from Thailand’s largest field hospital to emulate a target trial By emulation of a target trial where patients were hypothetically randomly assigned to Favipiravir with dexamethasone (FPV with dexa), Favipiravir alone (FPV alone), and symptomatic treatment (ST), our study strengthens the causal interpretation of the observed favipiravir efficacy. The findings suggest a modest survival benefit from Favipiravir-based therapies which demonstrated an average gain of 1–2 days of survival time. The results reflect clinically meaningful reductions in mortality risk over a large cohort, especially in a high-risk hospitalized population. Importantly, the direction of effects was consistent across sensitivity analyses among patients with particular prognosis factors, providing plausibility to therapeutic benefit of favipiravir.

To compare survival outcome of treatments, the emulated target trial methodology is particularly relevant to our context because traditional observational analyses are prone to bias. First, imbalanced prognostic characteristics between treatment arms can result in a baseline confounding bias. Second, different treatment initiation times significantly reflect the immortal time bias [20, 21]. Patients who were assigned to received FPV and end up in the treated arm must survive until the treatment initiation. This artificially contributes to an inflated beneficial effect of FPV if both symptomatic treatment and FPV groups were equally defined at time zero and followed up while FPV groups must survive until prescription and the actual follow up begin. By emulating a trial protocol, we specifically defined a time zero of follow-up at admission for all treatment groups and allowed a grace period to receive FPV for one day after admission. Then, we cloned the patients to tripled and randomized them to each arm, thereby balancing the baseline across all study arms. The cloned data were censored, and the survival time analysis were weighted by censoring probability. Even the propensity score matching studies were also observed in favipiravir efficacy research [22-24], our study provides additional bias adjustment through the cloning, and censoring. From our analysis, the baseline BMI, duration from PCR to admission, underlying except DM and hypertension, and vaccination status were well balanced after weighting, while the remaining were later adjusted in the RMST model. Although the emulating a target trial may not fully replace an actual randomized controlled trial, but in a pandemic setting, this approach offers a rigorous way to extract causal insights from real-world data with minimized baseline imbalance and immortal time bias when RCTs are limited or delayed [25].

A number of clinical trials and observational studies have reported controversial results of mortality rate among moderate to severe COVID-19 patients treated with favipiravir (S2 Table). The largest randomized trial to date, the open-label PIONEER trial with 503 patients, reported no significant difference in 28-day mortality between Favipiravir and standard care overall (10% vs 14% mortality; HR 0.74, p=0.24) [44]. However, that trial hinted at potential subgroup effects: in patients under 60 years old, favipiravir significantly improved outcomes, including a 66% reduction in the risk of progressing to mechanical ventilation or death (HR 0.34, p=0.02). While RCT from Shenoy et al. in Kuwait reported non-significant different of mortality [45], study from Kamali et al. from Iran showed significantly better results of favipiravir in critically ill patients [46]. Providing that this study has imbalance baseline oxygen saturation, but the improvement among FPV group who had mean oxygen saturation <80% was obvious. Consistently in a real-world study, though the analysis was unmatched, favipiravir monotherapy can reduce 28-day mortality risk in severe COVID-19 comparing to no-antiviral treatment (relative risk = 0.72; 95% CI 0.58–0.91; p = 0.006) [19]. Our results similarly revealed that in moderate-to-severe case, patients tend to benefit from favipiravir treatment comparing to standard of care or symptomatic treatment, especially in patients with hypoxia, but not patients in older age.

Furthermore, the inclusion of dexamethasone in our combination arm, FPV with dexa, warrants discussion. Dexamethasone is a cornerstone of COVID-19 therapy after the RECOVERY trial demonstrated a significant mortality reduction. Patients on mechanical ventilation or oxygen therapy had substantially lower death rates with Dexamethasone (e.g. 29.3% vs 41.4% on ventilator) whereas patients not needing oxygen had no benefit and a non-significant trend with steroids [15]. Our results demonstrate an added survival benefit when combine dexamethasone with favipiravir providing a trend in prolonging survival time and those were more pronounced in patients with pneumonia or hypoxia. Similar results can be observed in several trials [35, 47]. However, the effect of dexamethasone in our study was not large enough to provide significant improvement compared to FPV alone. This might be because favipiravir’s effect might hinder the with anti-inflammatory action of dexamethasone.

The observed effects of favipiravir and dexamethasone can be understood in light of their pharmacological actions. Favipiravir has broad activity against RNA viruses by inhibiting the viral RNA-dependent RNA polymerase of SARS-CoV-2 and was one of the first antivirals repurposed for COVID-19. By reducing viral load, favipiravir may shorten the period of high viral replication and potentially mitigate the direct cytopathic effects of the virus [27]. This results in a faster resolution of fever and pneumonia, and a lower likelihood that the infection triggers a severe inflammatory cascade [6, 28, 29]. Importantly, favipiravir’s efficacy seems more dependent on timing [16, 48]. In our moderate cases, favipiravir alone likely helped by curbing the virus during the acute phase, which is supported by the modest survival improvement and aligns with reports of quicker clinical improvement in other studies [28, 30]. In more severe cases, any antiviral effect of favipiravir would be adjunctive that a combination of favipiravir with other supportive therapy showed more favorable outcomes for COVID-19 patients [28]. This is biologically plausible because the ongoing viral load may continue in some patients driving inflammation and a markedly increase of inflammatory markers [14, 31, 32]. This is where Dexamethasone plays a crucial role. Dexamethasone is a potent glucocorticoid that downregulates the production of pro-inflammatory cytokines, collectively reducing the inflammation-mediated lung injury that characterizes acute respiratory distress syndrome in severe COVID-19 [32-34]. This can be inferred that dexamethasone benefits the critically ill or hypoxic patients, [15, 35] but should not be used in mild cases who were not receiving respiratory support where possible harm can be observed due to impaired viral clearance from immunosuppression [15, 35].

With that being said, however, current variants and outpatient therapies have shifted the treatment landscape Our study was conducted in the unique context of Thailand’s largest field hospital during the country’s third wave of the COVID-19 pandemic (May–September 2020), when the access to remdesivir was limited and favipiravir was used as the frontline antiviral. At that time, the Thai government primarily imported favipiravir, and secure our own favipiravir supply, producing generic favipiravir tablets domestically. Therefore, favipiravir became the most widely used antiviral for symptomatic COVID-19 patients with risk factors [41]. However, although available, remdesivir was reserved for select cases based on physician discretion, particularly for pregnant women in their first trimester to avoid teratogenic effects and patients unable to take oral medications due to absorption issues. This context reflects a setting where treatment decisions influenced by medication availability and evolving clinical practices. While these circumstances may differ from later phases of the pandemic, particularly with the emergence of new variants, expanded vaccination coverage, and broader access to alternative antivirals, they represent real-world conditions faced by resource-limited setting during critical surges. These setting-specific factors may limit the generalizability of our findings to other populations or healthcare systems. However, the use of an emulated target trial framework in our analysis enhances the internal validity and supports causal inference. Additionally, the observed survival benefit of favipiravir, particularly in hypoxic patients, is consistent with findings from other trials and observational studies. Though our results suggest modest benefit gains in survival time of favipiravir, it is in the same range as that reported for remdesivir and other antiviral agents such as remdesivir Lopinavir/ ritonavir (S3 table) [23, 29, 36-38]. When used in conjunction with anti-inflammatory agents like dexamethasone, it may improve outcomes. Our findings suggest that favipiravir can be a valuable alternative in settings where other antivirals are inaccessible. It might not be the first choice for outpatients but could be useful for severe hospitalized patients with low oxygen saturation.

**Comment 24:**
The Kaplan-Meier (KM) estimation graph reveals a surprising drop in survival probability within the FPV-alone and symptomatic treatment groups, where the majority of cases are presumed to involve moderate COVID-19. This unexpected result raises questions about potential underlying factors influencing survival in these groups. To provide greater clarity, I strongly recommend that the authors separate moderate COVID-19 cases from severe COVID-19 cases in their analysis. This stratification would allow for a more accurate interpretation of survival outcomes and ensure that the observed trends are not confounded by differing disease severities.
**--------------------------------------------------------------**

**Response**

Thank you for the concern in moderate and severe cases.

However, the proportions of severe case in each treatment are 18.88%, 10.2%, 33.94% in FPV+dexa, FPV and ST groups, respectively. The majority of populations are moderate cases. Therefore, we revised the analysis as we separate the more severe cases from the moderate group. As a result, the groups receiving anti-viral agent and dexamethasone provided a slightly more pronounced resutls that the group receiving FPV alone when compared to symtomatic treatment with RMST difference 2.83 (95%CI 0.81,4.86; p-value = 0.006) and 2.54 (95%CI 0.10,4.98; p-value =0.041). The resutls are illustrated in **S3 Table.**

**Comment 25:**
For covariates with very small, standardized differences after weighting (e.g., age, BMI, comorbidities), the results can be interpreted with higher confidence as these variables are well-balanced between groups.
Overall, I recommend the authors consult the medical statisticians and include them in coauthors to clarify and help improve residual imbalances and further enhance the validity of your study results.

**--------------------------------------------------------------**

**Response**

**We have added to the discussion as follows:**

To compare survival outcome of treatments, the emulated target trial methodology is particularly relevant to our context because traditional observational analyses are prone to bias. First, imbalanced prognostic characteristics between treatment arms can result in a baseline confounding bias. Second, different treatment initiation times significantly reflect the immortal time bias [20, 21]. Patients who were assigned to received FPV and end up in the treated arm must survive until the treatment initiation. This artificially contributes to an inflated beneficial effect of FPV if both symptomatic treatment and FPV groups were equally defined at time zero and followed up while FPV groups must survive until prescription and the actual follow up begin. By emulating a trial protocol, we specifically defined a time zero of follow-up at admission for all treatment groups and allowed a grace period to receive FPV for one day after admission. Then, we cloned the patients to tripled and randomized them to each arm, thereby balancing the baseline across all study arms. The cloned data were censored, and the survival time analysis were weighted by censoring probability. Even the propensity score matching studies were also observed in favipiravir efficacy research [22-24], our study provides additional bias adjustment through the cloning, and censoring. From our analysis, the baseline BMI, duration from PCR to admission, underlying except DM and hypertension, and vaccination status were well balanced after weighting, while the remaining were later adjusted in the RMST model. Although the emulating a target trial may not fully replace an actual randomized controlled trial, but in a pandemic setting, this approach offers a rigorous way to extract causal insights from real-world data with minimized baseline imbalance and immortal time bias when RCTs are limited or delayed [25].

**References**

1.	Health. NIo. COVID-19 Treatment Guidelines Panel 2024 [Available from: <https://www.covid19treatmentguidelines.nih.gov/>.

2.	Sohrabi C, Alsafi Z, O'Neill N, Khan M, Kerwan A, Al-Jabir A, et al. World Health Organization declares global emergency: A review of the 2019 novel coronavirus (COVID-19). Int J Surg. 2020;76:71-6.

3.	Agrawal U, Raju R, Udwadia ZF. Favipiravir: A new and emerging antiviral option in COVID-19. Med J Armed Forces India. 2020;76(4):370-6.

4.	Department of Medical Services T. COVID-19 Treatment Guidelines 21 March 2020. In: Department of Medical Services MoPH, editor. 2020.

5.	Narumol Sawanpanyalert RS, Piamlarp Sangsayunh, Opass Putcharoen, Weerawat Manosuthi, Poj Intalapaporn, Nattawan Palavutitotai,, Worawan Samritmanoporn NJ, Alan Maleesatharn, Chokephaibulkit aK. Assessement of outcomes following implementation of antiviral treatment guidelines for covid-19 during the first wave in thailand. Southeast Asian Journal of Tropical Medicine and Public Health. 2021;52(4):572-85.

6.	Shiraki K, Daikoku T. Favipiravir, an anti-influenza drug against life-threatening RNA virus infections. Pharmacol Ther. 2020;209:107512.

7.	Rattanachaikunsopon P PP. Third Wave of COVID-19 in Thailand.

International Journal of Current Research and Review. 2021;13(09).

8.	Tantipasawasin S. SARS-CoV-2 Delta Variant: The 2nd Wave COVID-19 Pandemic in Thailand. Chonburi Hospital Journal 2021;46(2):81-2.

9.	Twohig KA, Nyberg T, Zaidi A, Thelwall S, Sinnathamby MA, Aliabadi S, et al. Hospital admission and emergency care attendance risk for SARS-CoV-2 delta (B.1.617.2) compared with alpha (B.1.1.7) variants of concern: a cohort study. The Lancet Infectious Diseases. 2022;22(1):35-42.

10.	Department of Medical Services T. COVID-19 Treatment Guidelines 18 April 2023. In: Department of Medical Services MoPH, editor. 2023.

11.	Cai Q, Yang M, Liu D, Chen J, Shu D, Xia J, et al. Experimental Treatment with Favipiravir for COVID-19: An Open-Label Control Study. Engineering (Beijing). 2020;6(10):1192-8.

12.	Doi Y, Ishihara T, Banno S, Ando M, Kondo M. Favipiravir for symptomatic COVID-19: A nationwide observational cohort study. J Infect Chemother. 2023;29(2):150-6.

13.	Bosaeed M, Mahmoud E, Alharbi A, Altayib H, Albayat H, Alharbi F, et al. Favipiravir and Hydroxychloroquine Combination Therapy in Patients with Moderate to Severe COVID-19 (FACCT Trial): An Open-Label, Multicenter, Randomized, Controlled Trial. Infect Dis Ther. 2021;10(4):2291-307.

14.	Svanberg R, MacPherson C, Zucco A, Agius R, Faitova T, Andersen MA, et al. Early stimulated immune responses predict clinical disease severity in hospitalized COVID-19 patients. Commun Med (Lond). 2022;2:114.

15.	Horby P, Lim WS, Emberson JR, Mafham M, Bell JL, Linsell L, et al. Dexamethasone in Hospitalized Patients with Covid-19. N Engl J Med. 2021;384(8):693-704.

16.	Sirijatuphat R, Manosuthi W, Niyomnaitham S, Owen A, Copeland KK, Charoenpong L, et al. Early treatment of Favipiravir in COVID-19 patients without pneumonia: a multicentre, open-labelled, randomized control study. Emerg Microbes Infect. 2022;11(1):2197-206.

17.	Siripongboonsitti T, Muadchimkaew M, Tawinprai K, Issaranon O, Meepholkij W, Arttawejkul P, et al. Favipiravir treatment in non-severe COVID-19: promising results from multicenter propensity score-matched study (FAVICOV). Sci Rep. 2023;13(1):14884.

18.	Rattanaumpawan P, Jirajariyavej S, Lerdlamyong K, Palavutitotai N, Saiyarin J. Real-World Effectiveness and Optimal Dosage of Favipiravir for Treatment of COVID-19: Results from a Multicenter Observational Study in Thailand. Antibiotics (Basel). 2022;11(6).

19.	Srisubat A, Thanasitthichai S, Kongsaengdao S, Maneeton N, Maneeton B, Akksilp S. Effectiveness of Favipiravir monotherapy in the treatment of COVID-19: real world data analysis from Thailand. Lancet Reg Health Southeast Asia. 2023;11:100166.

20.	Hernán MA, Sauer BC, Hernández-Díaz S, Platt R, Shrier I. Specifying a target trial prevents immortal time bias and other self-inflicted injuries in observational analyses. J Clin Epidemiol. 2016;79:70-5.

21.	Maringe C, Benitez Majano S, Exarchakou A, Smith M, Rachet B, Belot A, et al. Reflection on modern methods: trial emulation in the presence of immortal-time bias. Assessing the benefit of major surgery for elderly lung cancer patients using observational data. Int J Epidemiol. 2020;49(5):1719-29.

22.	Al Sulaiman K, Aljuhani O, Korayem GB, Altebainawi AF, AlFaifi M, Nahari M, et al. When antivirals backfire: An evaluation of favipiravir's clinical outcomes in critically ill patients with COVID-19: A multicenter cohort study. J Infect Public Health. 2023;16(9):1492-9.

23.	Chavalertsakul K, Sutherasan Y, Petnak T, Thammavaranucupt K, Kirdlarp S, Boonsarngsuk V, et al. Remdesivir versus Favipiravir in Hospitalized Patients with Moderate to Severe COVID-19 Pneumonia: A Propensity Score-Matched Retrospective Cohort Study. Int J Gen Med. 2024;17:2163-75.

24.	Mutair AA, Shamou J, Alhumaid S, Layqah L, Ahmed GY, Thoyaja K, et al. Overview of clinical outcome and therapeutic effectiveness of Favipiravir in patients with COVID-19 admitted to intensive care unit, Riyadh, Saudi Arabia. J Infect Public Health. 2022;15(4):389-94.

25.	Janiaud P, Axfors C, Ioannidis JPA, Hemkens LG. Recruitment and Results Reporting of COVID-19 Randomized Clinical Trials Registered in the First 100 Days of the Pandemic. JAMA Netw Open. 2021;4(3):e210330.

26.	Funk MJ, Westreich D, Wiesen C, Stürmer T, Brookhart MA, Davidian M. Doubly robust estimation of causal effects. Am J Epidemiol. 2011;173(7):761-7.

27.	Peng Q, Peng R, Yuan B, Wang M, Zhao J, Fu L, et al. Structural Basis of SARS-CoV-2 Polymerase Inhibition by Favipiravir. Innovation (Camb). 2021;2(1):100080.

28.	Qomara WF, Primanissa DN, Amalia SH, Purwadi FV, Zakiyah N. Effectiveness of Remdesivir, Lopinavir/Ritonavir, and Favipiravir for COVID-19 Treatment: A Systematic Review. Int J Gen Med. 2021;14:8557-71.

29.	Tabarsi P, Vahidi H, Saffaei A, Hashemian SMR, Jammati H, Daraei B, et al. Favipiravir Effects on the Control of Clinical Symptoms of Hospitalized COVID-19 Cases: An Experience with Iranian Formulated Dosage Form. Iran J Pharm Res. 2021;20(4):1-8.

30.	Al-Muhsen S, Al-Numair NS, Saheb Sharif-Askari N, Basamh R, Alyounes B, Jabaan A, et al. Favipiravir Effectiveness and Safety in Hospitalized Moderate-Severe COVID-19 Patients: Observational Prospective Multicenter Investigation in Saudi Arabia. Front Med (Lausanne). 2022;9:826247.

31.	Kuri-Ayache M, Rivera-Cavazos A, Pérez-Castillo MF, Santos-Macías JE, González-Cantú A, Luviano-García JA, et al. Viral load and its relationship with the inflammatory response and clinical outcomes in hospitalization of patients with COVID-19. Front Immunol. 2022;13:1060840.

32.	Moore JB, June CH. Cytokine release syndrome in severe COVID-19. Science. 2020;368(6490):473-4.

33.	Harvey BJ. Molecular mechanisms of dexamethasone actions in COVID-19: Ion channels and airway surface liquid dynamics. Steroids. 2024;202:109348.

34.	Neyton LPA, Patel RK, Sarma A, Ansel KM, Christenson S, Adkisson M, et al. Distinct pulmonary and systemic effects of dexamethasone in severe COVID-19. Nature Communications. 2024;15(1):5483.

35.	Angermair S, Hardenberg JH, Rubarth K, Balzer F, Akbari N, Menk M, et al. In-hospital survival of critically ill COVID-19 patients treated with glucocorticoids: a multicenter real-world data study. Sci Rep. 2024;14(1):12138.

36.	Chen C, Zhang Y, Huang J, Yin P, Cheng Z, Wu J, et al. Favipiravir Versus Arbidol for Clinical Recovery Rate in Moderate and Severe Adult COVID-19 Patients: A Prospective, Multicenter, Open-Label, Randomized Controlled Clinical Trial. Front Pharmacol. 2021;12:683296.

37.	Lou Y, Liu L, Yao H, Hu X, Su J, Xu K, et al. Clinical Outcomes and Plasma Concentrations of Baloxavir Marboxil and Favipiravir in COVID-19 Patients: An Exploratory Randomized, Controlled Trial. Eur J Pharm Sci. 2021;157:105631.

38.	Solaymani-Dodaran M, Ghanei M, Bagheri M, Qazvini A, Vahedi E, Hassan Saadat S, et al. Safety and efficacy of Favipiravir in moderate to severe SARS-CoV-2 pneumonia. Int Immunopharmacol. 2021;95:107522.

39.	Kim DH, Uno H, Wei LJ. Restricted Mean Survival Time as a Measure to Interpret Clinical Trial Results. JAMA Cardiol. 2017;2(11):1179-80.

40.	Kowarik A, Templ M. Imputation with the R Package VIM. Journal of Statistical Software. 2016;74(7):1 - 16.

41.	nation T. Locally made Favipiravir tablets used for Covid treatment get FDA approval Bangkok, Thailand: The Nation; 2021 [Available from: <https://www.nationthailand.com/in-focus/40003319>.

42.	2021. Available from: <https://tdri.or.th/2021/05/the-third-wave-of-covid-19-policy-suggestions/>.

43.	Organization WH. Clinical management of COVID-19: living guideline, 18 August 2023. Geneva: World Health Organization; 2023. Available from: <https://www.who.int/publications/i/item/WHO-2019-nCoV-clinical-2023.2>.

44.	Shah PL, Orton CM, Grinsztejn B, Donaldson GC, Crabtree Ramírez B, Tonkin J, et al. Favipiravir in patients hospitalised with COVID-19 (PIONEER trial): a multicentre, open-label, phase 3, randomised controlled trial of early intervention versus standard care. Lancet Respir Med. 2023;11(5):415-24.

45.	Shenoy S, Munjal S, Youha SA, Alghounaim M, Almazeedi S, Alshamali Y, et al. Favipiravir In Adults with Moderate to Severe COVID-19: A Phase 3 Multicentre, Randomized, Double-Blinded, Placebo-Controlled Trial. medRxiv; 2021.

46.	Kamali A, Sarmadian H, Mahmoodiyeh B, Valibeik S, Farmani F, Bashirgonbadi Z. Evaluation of the effect of favipiravir on patients with COVID-19. J Family Med Prim Care. 2023;12(2):242-5.

47.	Mourad A, Thibault D, Holland TL, Yang S, Young AR, Arnold Egloff SA, et al. Dexamethasone for Inpatients With COVID-19 in a National Cohort. JAMA Netw Open. 2023;6(4):e238516.

48.	Hobbs FDR, Gbinigie-Thompson OA, Shanyinde M, Yu L-M, Harris V, Dorward J, et al. Favipiravir for COVID-19 in adults in the community in PRINCIPLE, an open-label, randomised, controlled, adaptive platform trial of short- and longer-term outcomes. Journal of Infection. 2024;89(4).
